# Research on dual-output port on-board charging system based on CLLC resonant converter

**DOI:** 10.1371/journal.pone.0279558

**Published:** 2023-02-24

**Authors:** Kai Zhou, Hao Song, Ningzhi Jin, Dongyang Sun

**Affiliations:** Engineering Research Center of Automotive Electronics Drive Control and System Integration, Ministry of Education, Harbin University of Science and Technology, Harbin, Heilongjiang, China; Wuhan University, CHINA

## Abstract

This paper proposes a dual-output port on-board charging system based on a CLLC resonant converter, which can realize the function of simultaneously charging high-voltage power batteries and low-voltage batteries. The system topology is composed of a front-stage PFC and a latter-stage CLLC resonant converter. The system uses a three-port transformer to couple the resonant converter, the high-voltage charging circuit, and the low-voltage charging circuit. The system also realizes soft switching through resonant components. According to the design requirements of the charging system, the control strategy of the dual-output ports is determined by analyzing the working mode and gain characteristics of the system and the dual closed-loop control structure is designed to realize the functions of power factor correction and wide-range voltage regulation. Simulation and experimental results verify the correctness of theoretical analysis and structural design.

## 1 Introduction

With the increasing prominence of global environmental issues and the increasing popularity of electric vehicles, many auto companies focus on the design and development of electric vehicles [1, 2]. At present, vehicle-mounted power batteries and their charging technology have attracted much attention. Vehicle-mounted power batteries determine the performance indicators of electric vehicles, and charging technology directly affects the performance of power batteries [3, 4].

**Fig 1:**
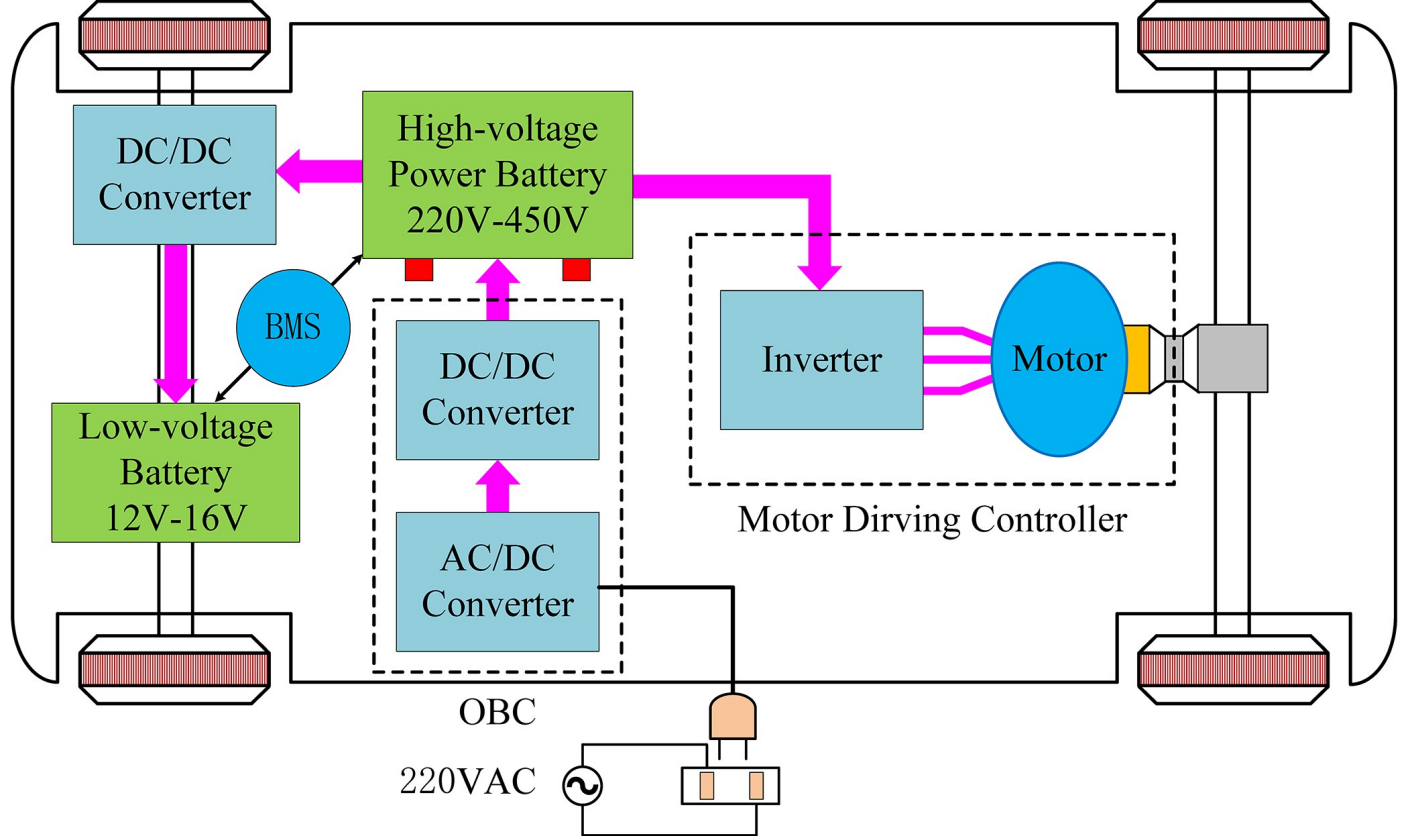
Typical electric structure of PEV.

Fig 1 shows the typical electric structure of pure electric vehicle (PEV), which mainly includes the on-board battery charger (OBC), motor drive controller and battery manage system (BMS), etc. As the core of energy conversion of electric vehicles, the OBC provides power for high-voltage power battery and low-voltage battery, which are detected and managed by BMS for charge quantity, while the motor driving part is controlled by motor driving controller [5].

As the key technology of pure electric vehicle drive system, the technical level of electric drive system directly determines the power and economy of PEV. At present, according to the layout of the drive system, PEV can be divided into centralized drive layout and distributed drive layout. The centralized drive system mainly uses the drive motor and reducer to replace the powertrain system of the traditional fuel vehicle to realize the electrification of the drive system, while the distributed drive is to install the drive motor directly on the wheel to drive the vehicle forward. Its structure and characteristics are shown in Table 1.

**Table 1:** Structure and characteristics of PEV.

Comprehensive performance	Centralized drive	Distributed drive
Number of motors	1 (Two wheel drive)	4 (Four wheel drive)
	2 (Four wheel drive)	
Number of inverters	1 (Two wheel drive)	4 (Four wheel drive)
	2 (Four wheel drive)	
Powertrain system	Automatic transmission	No gearbox
Battery voltage	About 400V	About 300V
Discharge of pollution	Zero emission	Zero emission
Main advantages	High maturity and popularity	Light weight and high efficiency
Main disadvantages	Poor system stiffness	Poor handling and protection
Scope of applications	Multiple types of passenger vehicles	No large-scale industrialization

As a device that is directly connected to the power grid, the on-board charging system mostly adopts a two-stage structure. The front-stage power factor correction (PFC) solves the interference problem of the charging system to the grid, suppresses the harmonics of input current, and provides a stable voltage for the later-stage DC bus. In order to adapt to the change of load during the charging process, the latter-stage DC-DC converter converts the constant voltage output by the front stage into a wide range of output voltage, and reduce the ripple of the output voltage for the front-stage PFC [6]. The front-stage PFC is generally a non-isolated converter, such as a Buck converter, a Boost converter, a derived bridgeless PFC, etc. [7]. The latter-stage DC-DC converter topology is divided into isolated converters and non-isolated converters. Isolated converters have received widespread attention because of their electrical isolation characteristics [8].

The high-voltage power battery charging circuit and the low-voltage battery charging circuit are important components of electric vehicles, but both sides are often operated separately, which have disadvantages such as large space occupation, high cost, and low efficiency. Domestic and foreign scholars have carried out research on the aforementioned problems. Mallik A proposed a Buck-Boost-based non-isolated dual-output port converter, which uses pulse width modulation (PWM) to achieve wide output voltage gain. However, it uses a high number of components and there is a problem in that the power switches are hard-switching, which causes a large switching loss [9]. Chen G proposed a non-isolated dual output port converter based on Cuk, which only uses two power switches, two diodes, three inductors, and three capacitors. The number of components used is reduced, but the problem of large switching loss in the power switches is introduced [10]. Prabhakaran P proposed a dual-input, dual-output non-isolated converter, which uses only one inductance element, which greatly reduces the volume of the converter, but both the current stress and switching loss are greater [11]. In order to reduce the switching loss of the power switches, Wu H proposed isolated full-bridge three-port converters, which uses PWM control to achieve zero voltage switching (ZVS) of the power switches on the primary side of the transformer. The switching loss of the power switches is reduced, but the converter gain range is lower [12]. Dao N proposed an isolated three-port converter based on series resonance and dual active bridge (DAB) structure, using a control strategy that combines phase-shift and frequency modulations to achieve wide output voltage gain, but its control strategy is more complicated and uses more components [13]. Oluwasogo E S adopted a self-current balancing structure of the dual-transformer-based triple-port active bridge (DT-DAB) converter. In comparison to the traditional DAB, it reduces the number of components, and adds an additional degree of freedom (DOF) on the primary side of H bridge, which expands the converter operation range and realizes ZVS of all power switches. However, increasing the amount of control increases the difficulty of system control and makes the control strategy more complicated [14]. Zhou K proposed a dual output port converter, which integrates high and low voltage circuits in an LLC resonant circuit and achieves a wide range of voltage output through dual closed-loop PI control. However, the low-voltage side of the circuit is an ordinary full-bridge circuit and does not have soft switching characteristics [15].

This paper proposes a dual-output port on-board charging system based on a CLLC resonant converter. The front-stage adopts a dual-diode bridgeless PFC, which improves the power factor while reducing common mode interference and outputs a stable DC bus voltage on latter-stage. The latter-stage integrates the high-voltage charging circuit with the low-voltage charging, and is coupled through a three-port transformer to meet the requirements of high efficiency and high power density of the on-board charging system [16]. In order to reduce switching loss and improve the efficiency of charging and discharging, the main topology adopts the CLLC resonant converter. Compared with the traditional LLC resonant converter, the CLLC resonant converter adds resonant components on the secondary side to ensure that all power switches can achieve ZVS during the battery charging process and battery discharge process, and further reduce the switching loss [17]. The control strategy adopts double closed-loop PI control to realize the power factor correction function of the front-stage circuit and the function of high-efficiency and wide-range output voltage of the latter-stage [18].

## 2 Structure of the on-board charging system

**Fig 2:**
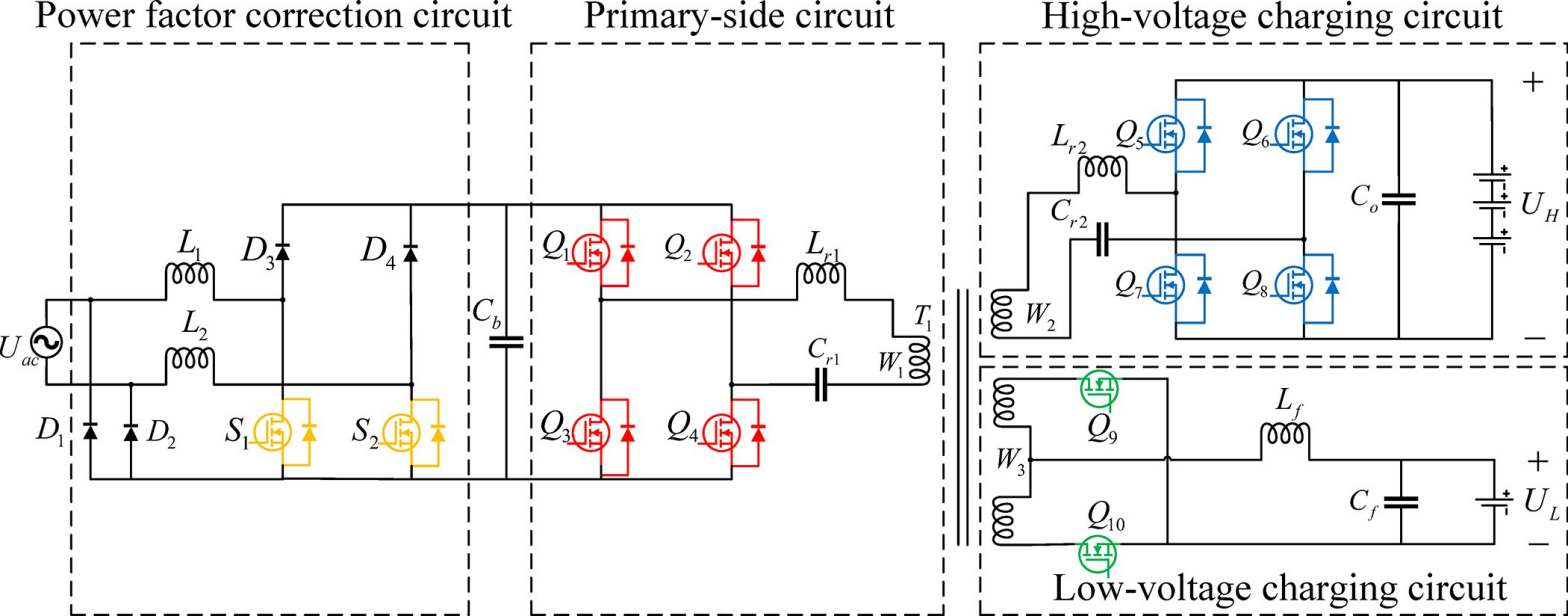
The topological structure of the dual-output on-board charging system.

Fig 2 shows the proposed topological structure of the dual-output on-board charging system, which includes a transformer primary-side circuit connected to a PFC circuit, a high-voltage charging circuit connected to a high-voltage power battery, and a low-voltage charging circuit connected to a low-voltage battery. The primary side circuit, high-voltage charging circuit, and low-voltage charging circuit are coupled through a transformer.

### 2.1 Power factor correction circuit

The power factor correction circuit primarily consists SiC-MOSFET S_1_ and S_2_, Schottky diodes D_1_~D_4_, DC bus capacitor C_b_, and two energy storage inductors L_1_ and L_2_. The front-stage circuit adopts a double-diode bridgeless PFC, and the input AC voltage source (*V*_ac_) are connected to D_1_ and D_2_. During operation of the circuit, there are only two semiconductor devices in the current flow path, so the switching loss is small, the efficiency is high. D_1_ and D_2_ connect the ground on the DC bus to the grid side, which can effectively suppress the generation of common mode interference, reduce the total harmonic distortion (THD) on the input side of the rectifier network, and provide a stable DC bus voltage for the latter-stage.

### 2.2 Primary-side circuit

The primary-side circuit primarily consists of MOSFET Q_1_~Q_4_, resonant inductor L_r1_, resonant capacitor C_r1_, and transformer primary winding W_1_, Q_1_~Q_4_ form a full-bridge circuit, L_r1_ and C_r1_ form a resonant circuit. The full-bridge circuit and the resonance circuit are resonant for converting the front-stage circuit of output DC bus voltage into high frequency voltage pulses and pass the high-frequency voltage pulse to the high voltage charging circuit and the low-voltage charging circuit through the winding W_1_.

### 2.3 High-voltage charging circuit

The high-voltage charging circuit primarily consists of MOSFET Q_5_~Q_8_, resonant inductor L_r2_, resonant capacitor C_r2_, sustaining capacitor C_0_, and transformer secondary winding W_2_, Q_5_~Q_8_ form a full-bridge circuit, L_r2_ and C_r2_ from a resonant circuit, and the output port of the circuit is connected to the high-voltage power battery. When the high-voltage power battery is in the charging state, the full-bridge circuit and the resonant circuit are responsible for converting the high-frequency voltage pulse output by the winding W_2_ into a DC voltage, and by maintaining capacitor C_0_ to ensure that the output voltage is stable. When in the discharging state, the full-bridge circuit and resonance circuit convert the output voltage of the high-voltage power battery into high-frequency voltage pulses and pass the high-frequency voltage pulses to the low-voltage charging circuit through the winding W_2_.

### 2.4 Low- voltage charging circuit

The low-voltage charging circuit primarily consists of MOSFET Q_9_ and Q_10_, filter inductor L_f_, filter capacitor C_f_, and transformer secondary winding W_3_. Q_9_ and Q_10_ form a full-wave rectifier circuit, L_f_ and C_f_ form a filter circuit. The output of the circuit is connected to the low voltage battery. The full-wave rectifier circuit converts the high-frequency voltage pulse output by winding W_3_ into a DC voltage, and filters high-order harmonics through a filter circuit to ensure stable output voltage.

### 2.5 Operating mode

The system has two working modes: parked charging mode and driving charging mode.

**Fig 3:**
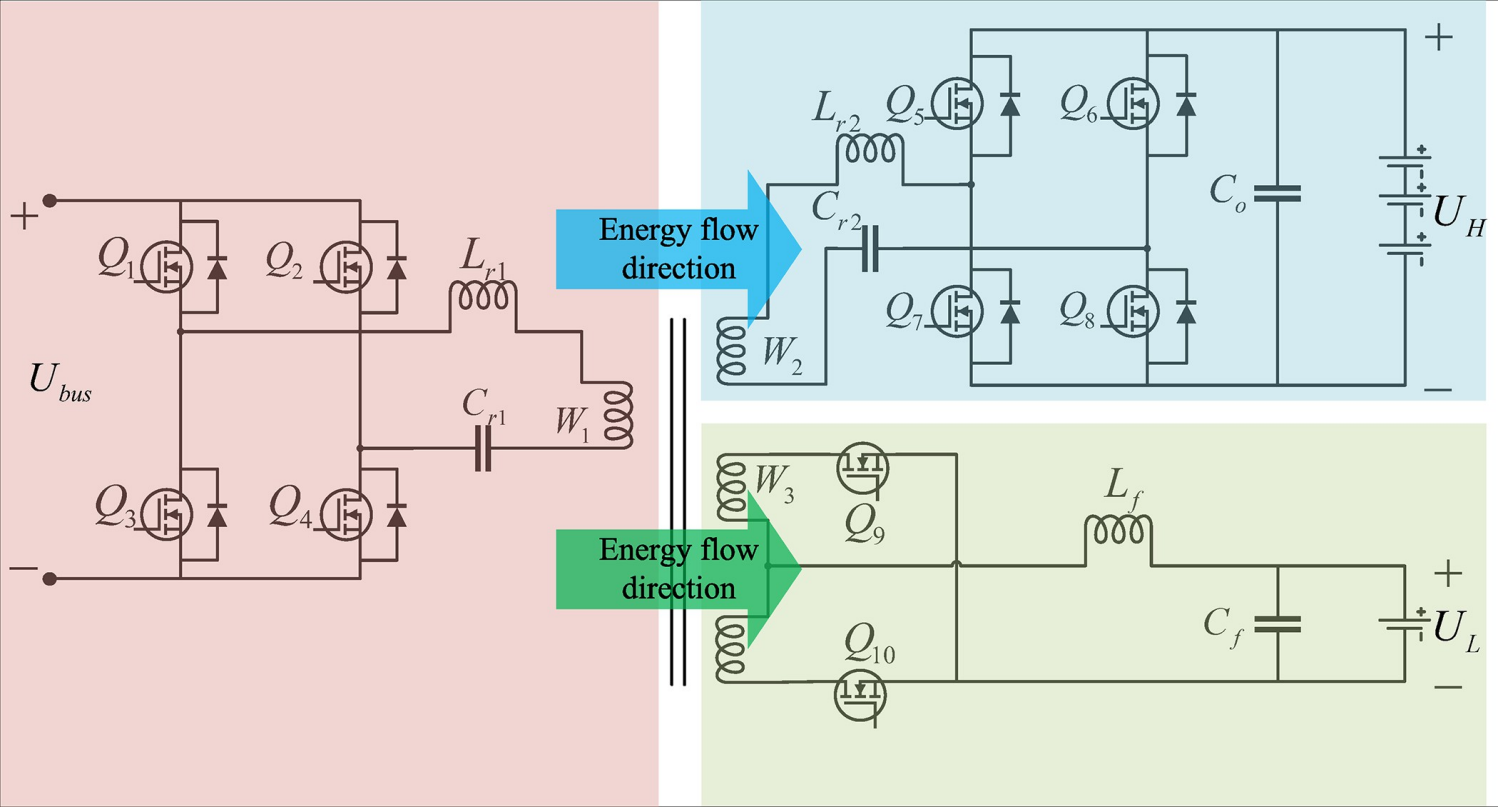
Parked charging mode.

Fig 3 shows the parked charging mode. When the BMS detects that the power of the high voltage power battery is insufficient, the switching frequency of Q_1_~Q_4_ is stable near the resonant frequency and Q_5_~Q_8_ are in the synchronous rectification working state. At this time, the energy is transferred from the primary-side circuit to the high-voltage charging circuit through T_1_ to charge the high-voltage power battery. When the BMS detects that the low-voltage battery is low, the switching frequency of Q_1_~Q_4_ is stable near the resonant frequency, where Q_9_ and Q_10_ are in the synchronous rectification state. At this time, the energy is transferred from the primary-side circuit to the low-voltage charging circuit through T_1_ to charge the high-voltage power battery. The external control circuit adjusts the frequency of Q_1_~Q_4_ to realize the regulation of the output voltage of the high-voltage charging circuit and the low-voltage charging circuit.

**Fig 4:**
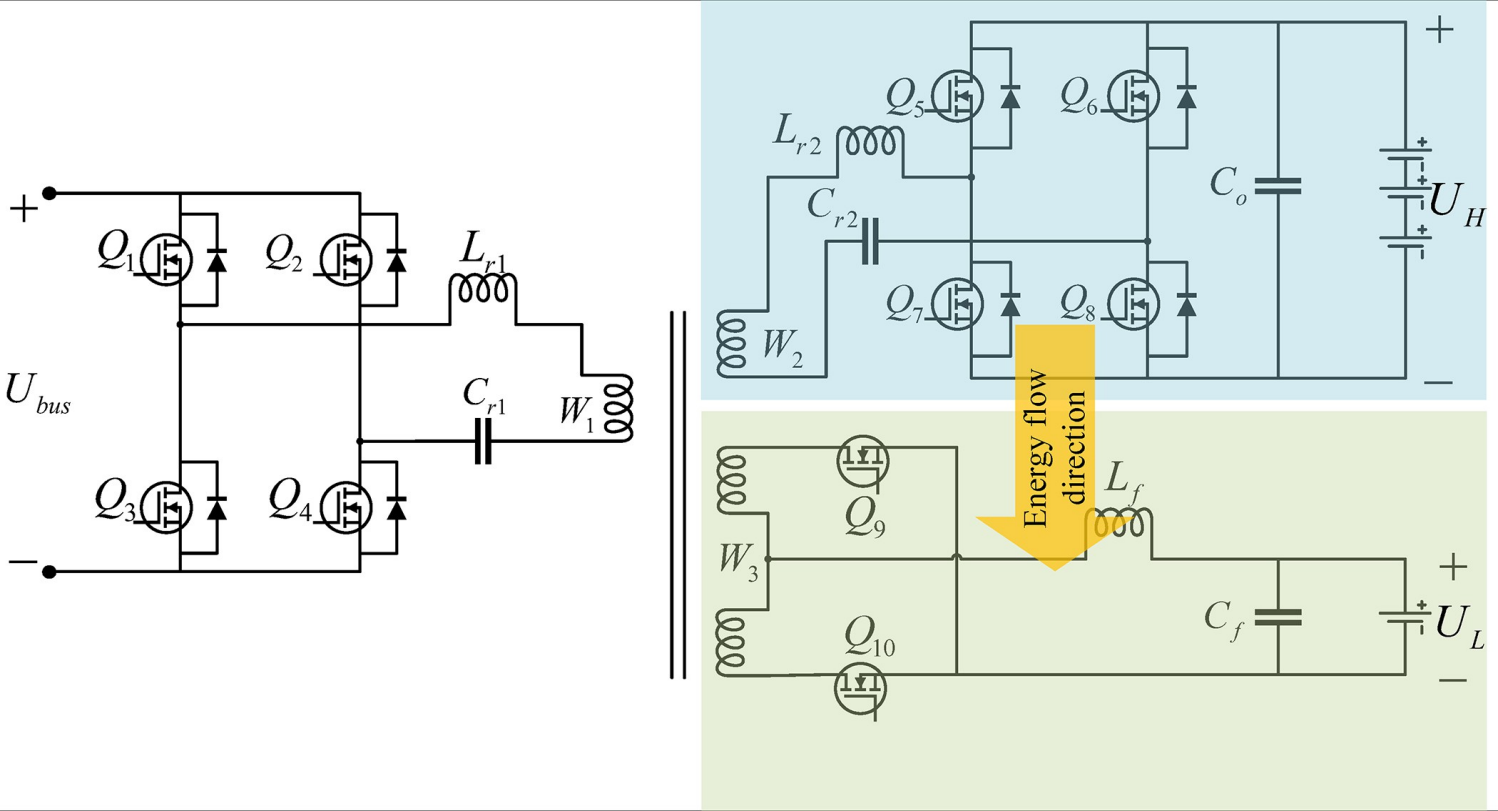
Driving charging mode.

When the system works in the driving charging mode, Q_1_~Q_4_ are in the off state. At this time, the high-voltage charging circuit and the low-voltage charging circuit form a series resonant circuit, as shown in Fig 4. When the BMS detects that the low-voltage battery has insufficient power, the switching frequency of Q_5_~Q_8_ is stable near the resonant frequency, where Q_9_ and Q_10_ are in the synchronous rectification state. At this time, energy is transferred from the high voltage charging circuit to the low-voltage charging circuit through T_1_ to charge the low-voltage battery. The external control circuit uses pulse frequency modulation (PFM) to control the frequency of Q_5_~Q_8_ to adjust the output voltage of the low-voltage charging circuit.

## 3 The voltage gain characteristics of the system

**Fig 5:**
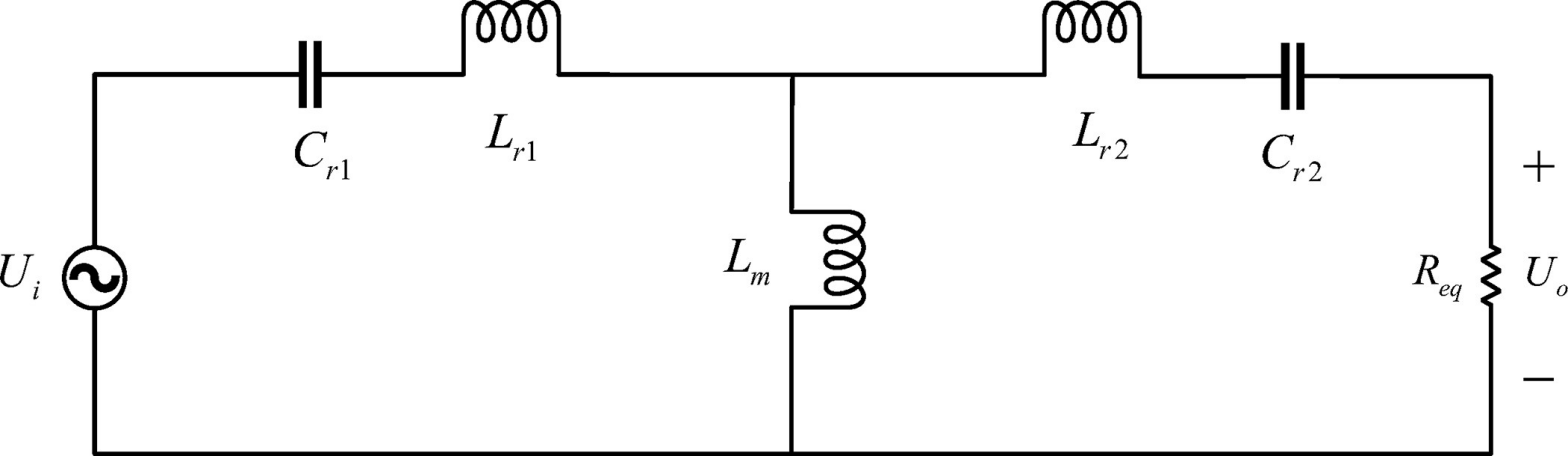
FHA method.

Fig 5 shows the equivalent model of the CLLC resonant converter based on the fundamental harmonic analysis (FHA) method. *R*_eq_ is the equivalent resistance of the high-voltage side converted to the primary side, and its equation is as follows:

$$R_{eq}=\frac{8n^{2}}{\pi^{2}}\frac{U_{out}}{I_{out}}=\frac{8n^{2}}{\pi^{2}}\frac{U_{out}^{2}}{P_{out}}$$ (1)

where, *n* is the transformer turn ratio, *U*_out_, *I*_out_ and *P*_out_ are the output voltage, current and power of the high-voltage side, respectively.

Make the CLLC converter resonant component parameters symmetrical, as shown below:

$$\left\{ \begin{array}{l} L_{r2}=\frac{L_{r1}}{n^{2}}\\ C_{r2}=n^{2}C_{r1} \end{array}\right.$$ (2)

where, *L*_r1_ and *C*_r1_ are the primary-side resonant inductor and resonant capacitor, *L*_r2_ and *C*_r2_ are the secondary side resonant inductors and resonant capacitors.

The DC voltage gain characteristics of the CLLC resonant converter are obtained as follows:

$$M(f_{n},Q,k)=\frac{1}{\sqrt{{\left(1+\frac{1}{k}-\frac{1}{kf_{n}}\right)}^{2}+\frac{Q^{2}}{k^{2}}{\left[(2k+1)f_{n}-\frac{2k+2}{f_{n}}+\frac{1}{f_{n}^{3}}\right]}^{2}}}$$ (3)

where, *M* is voltage gain, *f*_n_ is the normalized frequency, *Q* is the quality factor, and *k* is the inductance coefficient.


$$f_{n}=\frac{f_{s}}{f_{r1}}=2\pi f_{s}\sqrt{L_{r1}C_{r1}}$$ (4)

Here, *f*_r1_ is the resonant frequency of the converter, its value is 100 kHz, and *f*_s_ is the actual switching frequency.


$$k=\frac{L_{m}}{L_{r1}}=\frac{L_{m}}{n^{2}L_{r2}}$$ (5)

Here, *L*_m_ is the magnetizing inductor.


$$Q=\frac{\sqrt{L_{r1}/C_{r1}}}{R_{eq}}=\frac{\pi^{2}}{8}\frac{P_{out}}{{\left(nU_{out}\right)}^{2}}\sqrt{\frac{L_{r1}}{C_{r1}}}$$ (6)

**Fig 6:**
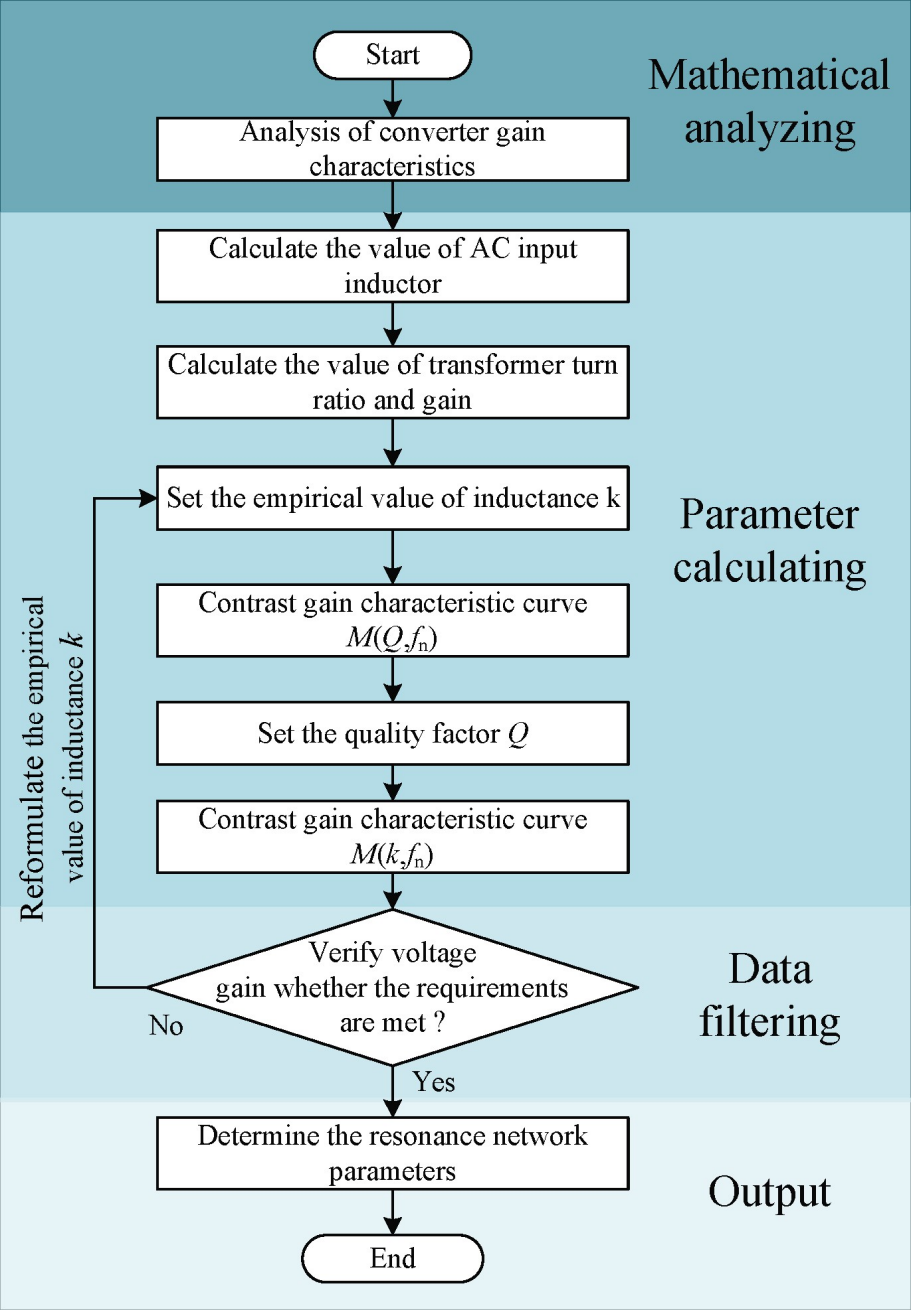
Design process of system parameters.

From Eq (3), the converter voltage gain expression contains three parameters, and direct analysis will lead to redundant parameter design and complicated analysis process. This paper proposes a simplified analysis method, through to formulate a parameter values for the analysis of the other two parameters and the relationship between the voltage gain,. Since *k* is relatively easy to determine compared with *Q* and *f*_n_, this paper firstly uses the provisional *k* value to analyze the change relationship between *M* and *f*_n_ and *Q*, then the provisional *Q* value to analyze the change relationship between *M* and *f* and *k*, and finally determines the appropriate *k* value and *Q* value by combining the analysis of the two. The specific parameter design process is shown in Fig 6.

**Fig 7:**
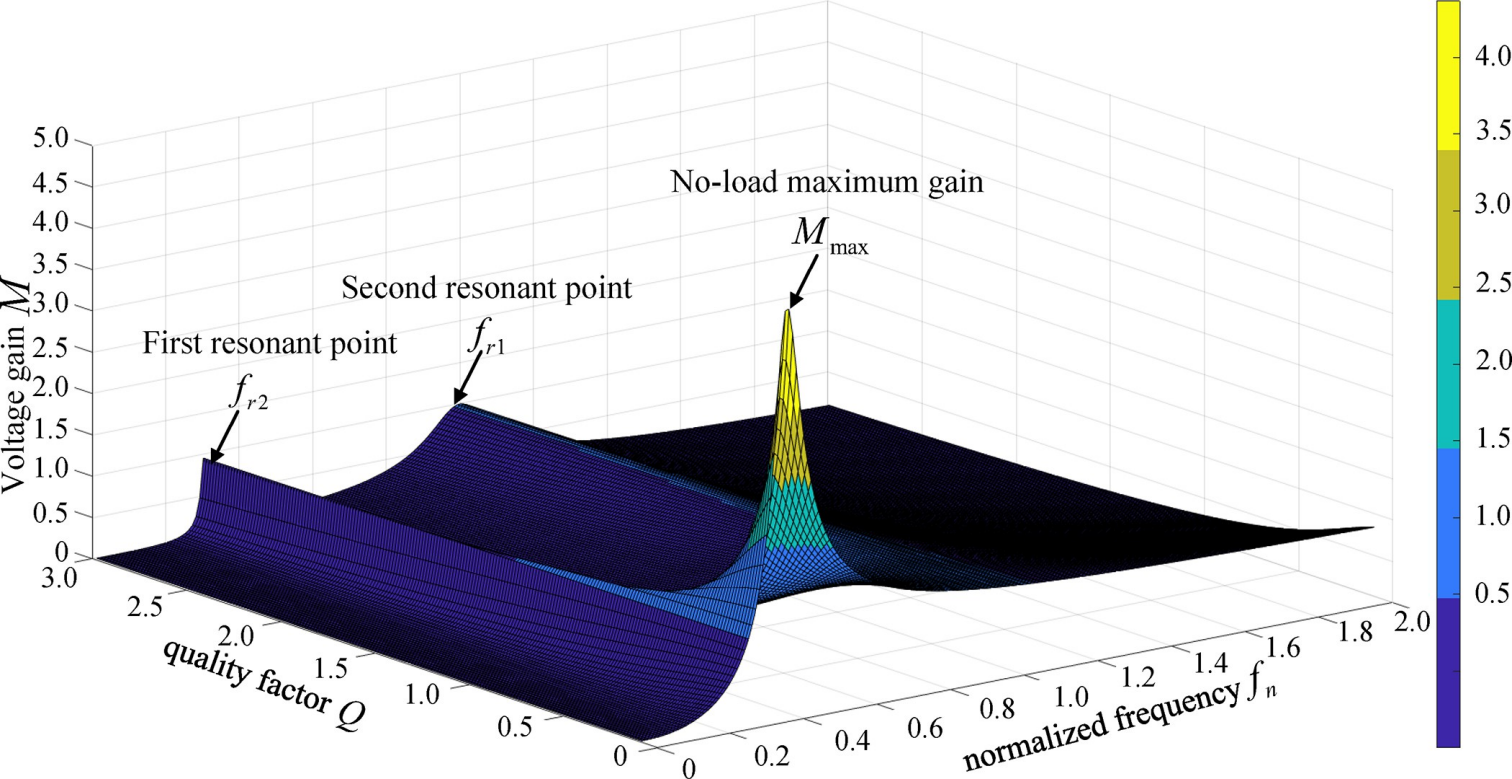
Three-dimensional surface plot of voltage gain characteristics.

The usual empirical value of inductance is 1 to 7, and the larger the value of k, the smaller the resonant current value and the smaller the loss. However, a larger value of *k* will widen the frequency range of the converter and reduce the service life of magnetic components. The empirical value of inductance is usually chosen as 5 in engineering, so k = 5 is drawn up in this paper, and the relationship between converter voltage gain *M*, *f*_n_ and *Q* can be obtained, as shown in Fig 7.

Fig 7 shows that there are two peaks in voltage gain *M* and when *Q* = 0 (under no-load state), there is a maximum gain *M*_max_. Thus, there are two resonant frequencies that affect the change of the gain curve. Define the second resonance frequency as *f*_r2_ as follows:

$$f_{r2}=\frac{1}{2\pi \sqrt{(L_{r1}+L_{m})L_{r1}}}=\frac{f_{r1}}{\sqrt{k+1}}$$ (7)

Define the normalized first resonant frequency and second resonant frequency as *f*_n1_ and *f*_n2_ as follows:

$$\left\{ \begin{array}{l} f_{n1}=\frac{f_{r1}}{f_{r1}}=1\\ f_{n2}=\frac{f_{r2}}{f_{r1}}=\frac{1}{\sqrt{k+1}} \end{array}\right.$$ (8)

**Fig 8:**
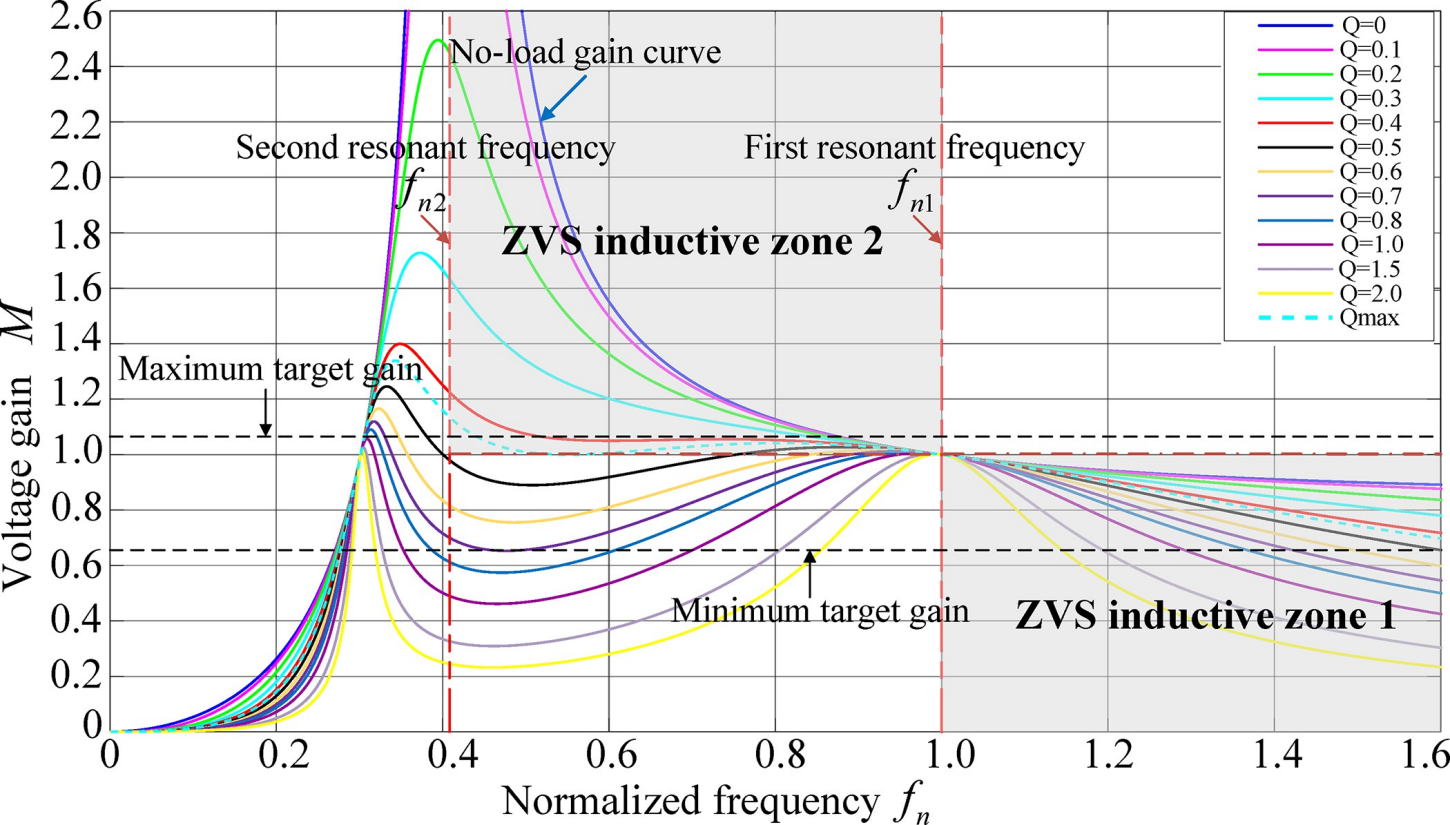
Voltage gain characteristics.

When *k* = 5, the influence of two resonant frequencies and quality factor *Q* on the gain curve is shown in Fig 8.

Fig 8 shows that the larger the value of *Q*, the smaller the gain, and the lower the frequency sensitivity. The smaller the value of *Q*, the greater the gain and the higher the frequency sensitivity, but it is unstable. In order to realize the accuracy of closed-loop voltage regulation control, it is necessary to ensure that the voltage gain decreases monotonously as the frequency increases during the working process of the converter. At the same time, in order to meet the soft switching conditions, the circuit should be in an inductive working state. The key is that the input impedance is inductive. For the CLLC resonant converter, its input impedance expression is as follows:

$$Z_{in}=Z_{r1}+Z_{m}∥(Z_{r2}+R_{eq})=j\omega L_{r1}+\frac{1}{j\omega C_{r1}}+\frac{j\omega L_{m}(j\omega C_{r1}R_{eq}-\omega^{2}L_{r1}C_{r1}+1)}{j\omega C_{r1}R_{eq}-\omega^{2}C_{r1}(L_{r1}+L_{m})+1}$$ (9)

Let the imaginary part of *Z*_in_ be 0. Then the maximum quality factor *Q*_max_ of the circuit working in the inductive range is as follows:

$$Q_{\max}=\frac{f_{n}}{\sqrt{\left(1-f_{n}^{2})[(2k+1)f_{n}^{2}-1\right]}}$$ (10)

The minimum value is as follows:

$$Q_{\max|f_{n}=\frac{1}{\sqrt[{4}]{2k+1}}}=\frac{1}{{\left(2k+1\right)}^{1/4}\sqrt{\left(1-\frac{1}{\sqrt{2k+1}})(\sqrt{2k+1}-1\right)}}$$ (11)

When *k* = 5, the minimum value of *Q* is 0.437.

**Fig 9:**
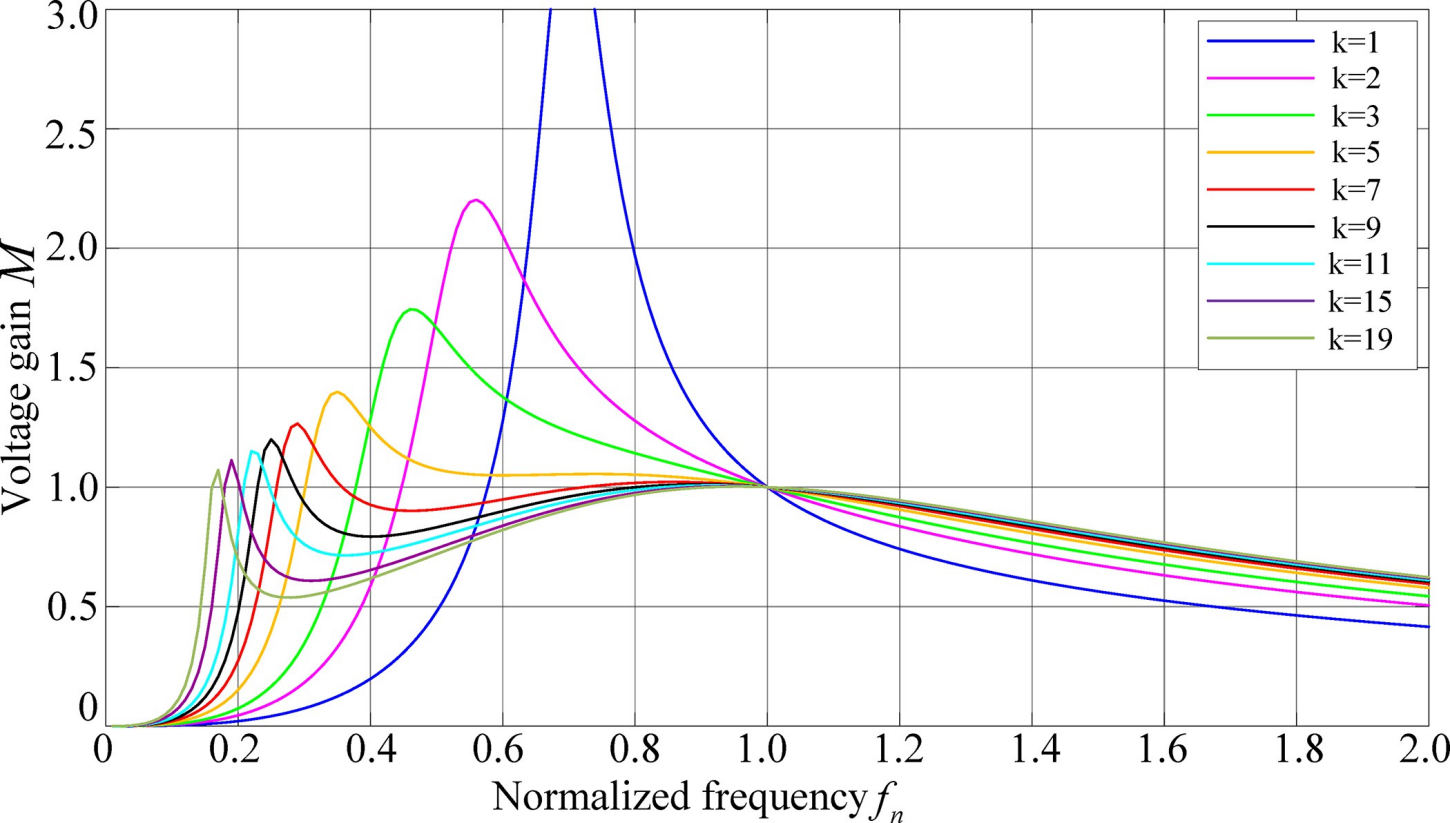
The influence of inductance coefficient on voltage gain characteristics.

In order to weigh the influence of the inductance coefficient on the voltage gain, when *Q* = 0.3, the relationship curve between the converter voltage gain *M*, *f*_n_ and *Q* can be obtained, as shown in Fig 9.

Fig 9 show that as the value of *k* decrease, its maximum gain range increases and the frequency sensitivity also increases, but a smaller *k* will make gain stability worse. A larger value of *k* will result in insufficient gain or a non-monotonous decreasing trend of voltage gain with increasing frequency, which increases the difficulty of closed-loop control.

## 4 System parameters design

Table 2 shows the design parameters of the dual-output port on-board charging system.

**Table 2:** Design metrics for integrated controllers.

Parameter	Value
AC input voltage	220 VAC
DC bus voltage	400 VDC
High-voltage rated output power	3.3 kW
Low-voltage rated output power	1.5 kW
High-voltage output range	300–400 V
Low-voltage output range	9–16 V
Resonant frequency	100 kHz
Power factor	≥0.99
Efficiency	≥95%

### 4.1 AC input inductor

Since the switching frequency is much higher than the input voltage frequency in the same switching cycle, it can be assumed that the input voltage does not change, and the circuit can be regarded as a Boost converter. When the power switches are turned on, the calculation formula of the inductor voltage is as follows:

$$U_{in}=L\frac{\Delta I}{\Delta t}=L\frac{\Delta I}{T_{sw}D}$$ (12)

Here, *U*_in_ is the input voltage, *D* is the duty cycle, *T*_sw_ is the switching period, Δ*I* is the inductor current ripple, and the ripple coefficient should not be greater than 20%, which can be obtained as follows:

$$\Delta I\leq 0.2I_{p}=\frac{0.2P}{\eta U_{bus}(1-D)}$$ (13)

Here, *I*_p_ is the peak value of the input current, *P* is the rated power, *U*_bus_ is the output DC bus voltage, *η* is the working efficiency of the PFC, generally not lower than 0.95 under full load conditions, and the inductor *L* is obtained by incorporating the system design parameters as follows:

$$L>5\frac{\eta {\left(1-D\right)}^{2}U_{bus}{}^{2}}{P}DT_{sw}=341.2\mu H$$ (14)

### 4.2 Transformer ratio

According to the design requirements of the system parameters, the converter voltage gain must be the largest when the DC bus voltage is 400V and the high voltage output is 400V. The ratio *n*_1_ of the transformer primary winding *W*_2_ to the high voltage side winding as follows:

$$n_{1}=\frac{U_{bus\_\max}}{U_{H\_\max}}=\frac{400}{400}=1$$ (15)

where, *U*_bus_max_ is the maximum value of DC bus voltage and *U*_H_max_ is the maximum value of high-voltage output.

In order to ensure that the low-voltage electrical equipment in the car can operate normally when the high-voltage power battery is low, the transformation ratio *n*_2_ of the high voltage side winding and the low voltage side winding *W*_3_ is as follows:

$$n_{2}=\frac{U_{H\_\min}}{U_{L\_\max}}=\frac{300}{16}=18.75$$ (16)

where, *U*_H_min_ is the minimum output voltage on the high-voltage side and *U*_L_max_ is the maximum output voltage on the low-voltage side.

### 4.3 Maximum gain and minimum gain

When the system charges the vehicle power battery, its maximum gain *M*_H_max_ and minimum gain *M*_H_min_ are as follows:

$$M_{H\_\max}=\frac{n_{1}(U_{H\_\max}+U_{of})}{U_{bus\_\min}}=1.0621$$ (17)

$$M_{H\_\min}=\frac{n_{1}(U_{H\_\min}+U_{of})}{V_{bus\_\max}}=0.671$$ (18)

where, *U*_H_max_ is the maximum output voltage on the high voltage side, *U*_bus_min_ is the minimum input voltage on the DC bus side, and *U*_of_ is the switch voltage drop (*U*_of_ = 1V).

When the system is charging the low-voltage battery on the vehicle, its maximum gain *M*_L_max_ and minimum gain *M*_L_min_ are as follows:

$$M_{L\_\max}=\frac{n_{2}(U_{L\_\max}+U_{of})}{U_{H\_\min}}=1.079$$ (19)

$$M_{L\_\min}=\frac{n_{2}(U_{L\_\min}+U_{of})}{U_{H\_\max}}=0.704$$ (20)

where, *U*_L_min_ is the minimum output voltage on the low-voltage side and *U*_of_ is the power switch voltage drop (*U*_of_ = 1V).

### 4.4 Resonant inductor, resonant capacitor, and magnetizing inductor

It can be deduced from Figs 8 and 9 that the inductance coefficient *k* satisfies the system’s requirements of maximum gain, minimum gain, and the monotonicity of gain, At this time, it is necessary to ensure that the circuit operates in ZVS inductive zones 1 and 2 and the quality factor *Q* is not greater than *Q*_max_ (0.437). However, the larger the value of *Q*, the lower the frequency sensitivity and the better the stability. Therefore, the value of *Q* should be as large as possible and the value of *Q* should be 0.42 according to the design.

From Eqs (5) and (6), the calculation formulas for resonant inductor, capacitance, and magnetizing inductor are as follows:

$$L_{r1}=\frac{QR_{eq}}{2\pi f_{r1}}=26.317\mu H$$ (21)

$$C_{r1}=\frac{1}{2\pi f_{r2}QR_{eq}}=96.175\mathrm{nF}$$ (22)

$$L_{m}=kL_{r1}=131.539\mu H$$ (23)

$$L_{r2}=\frac{L_{r1}}{n_{1}^{2}}=26.317\mu H$$ (24)

$$C_{r2}=n_{1}{}^{2}C_{r1}=131.539\mathrm{nF}$$ (25)

In order to meet the ZVS of the power switches, the dead time is set to 200ns.

## 5 System control strategy

**Fig 10:**
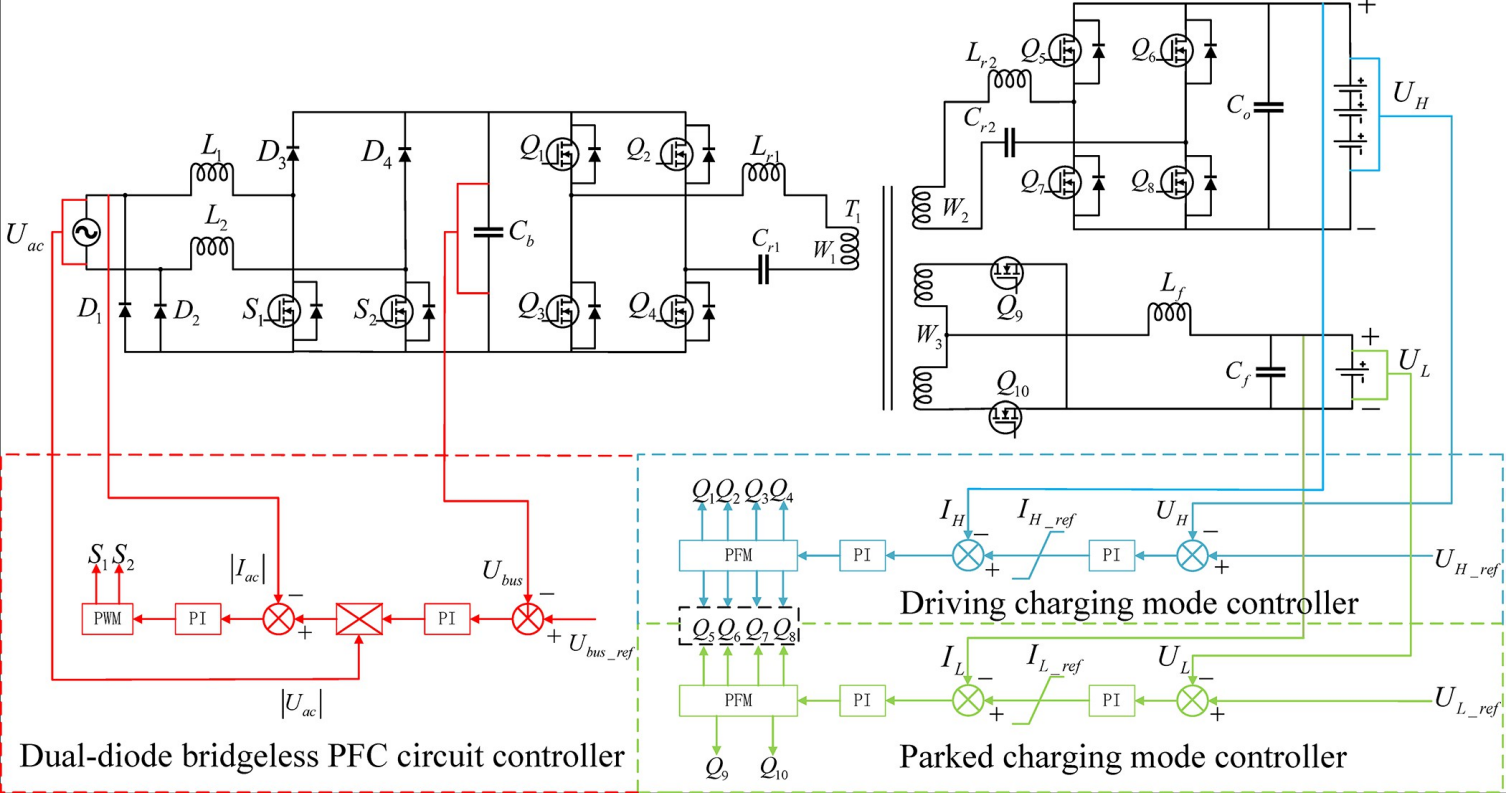
System control strategy.

Fig 10 shows the control strategy of the dual output port vehicle charging system. The control part mainly includes a dual-diode bridgeless PFC circuit controller, a parked charging mode controller and a driving charging mode controller.

The dual-diode bridgeless PFC controller adopts double closed-loop control based on the average current method. Its voltage outer loop is the difference between the output bus voltage (*U*_bus_) of the previous stage and the reference voltage (*U*_bus_ref_). After PI adjustment, it is multiplied with the modulus of the input voltage (|*U*_ac_|) and finally the current inner loop reference voltage is obtained. The current inner loop is the difference between the reference current and the modulus of input current (|*I*_ac_|). The duty cycle signal is obtained through PI adjustment. The duty cycle of *S*_1_ and *S*_2_ is controlled by PWM to realize the power factor correction function.

**Fig 11:**
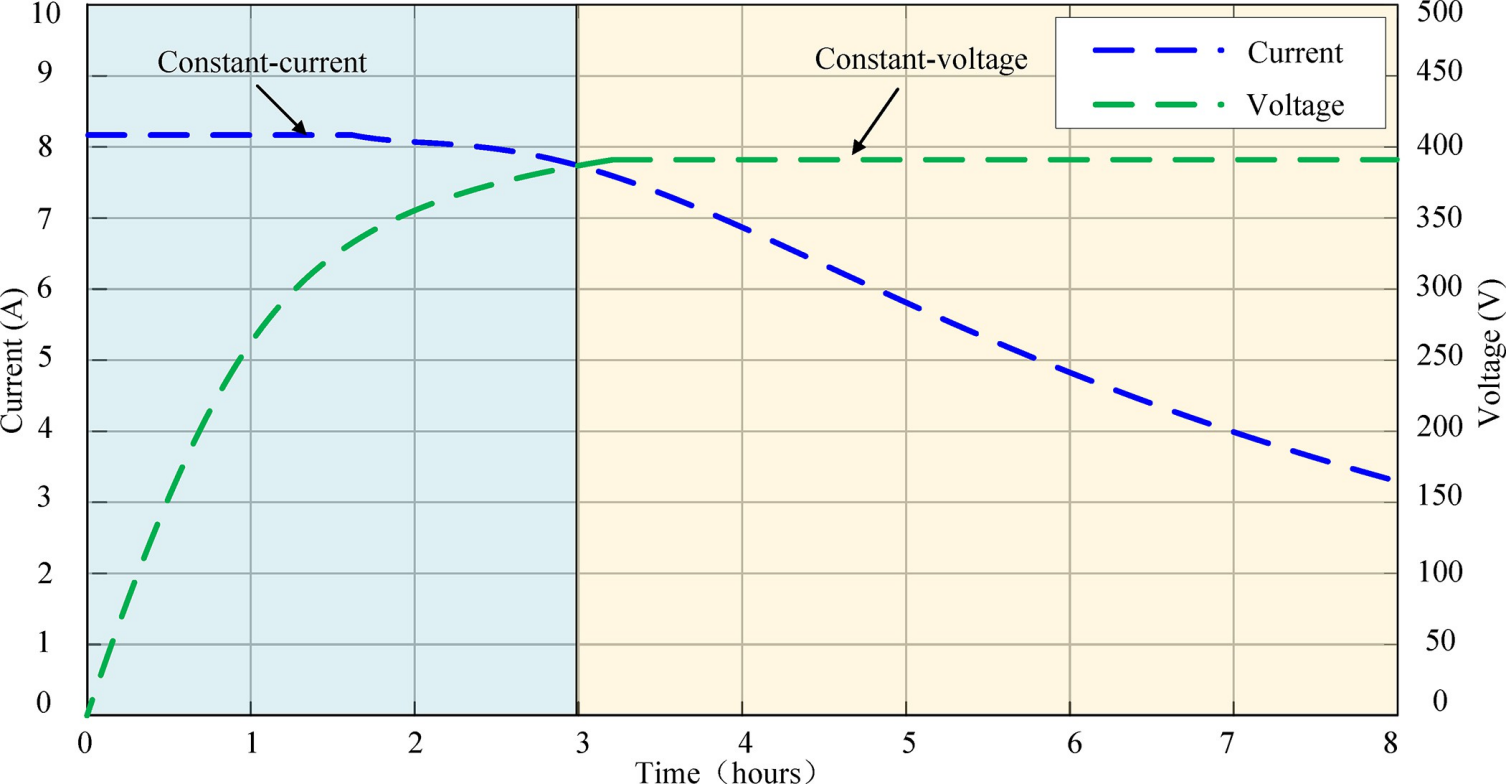
The effect of CC/CV charging strategy.

Both the parked and the driving charging mode controllers adopt voltage-current double closed-loop control. The output voltage and current are introduced as the control variables of the voltage outer loop and the current inner loop for PI adjustment and the power switches frequency is controlled by PFM. Therefore, the adjustment function of the output voltage is realized. Its charging mode adopts the strategy of constant-current constant-voltage (CC/CV) and uses the limiting secondary link to limit the output value of the voltage outer loop. That is, the voltage outer loop output value is greater than *I*_H_ref_ in the initial stage, then the reference value of the outer loop output circuit is limited to *I*_H_ref_. Therefore, the constant current charging is realized. As the voltage rises, the output value of the voltage outer loop is less than *I*_H_ref_. At this time, the output current reference value of the outer loop is input to the current inner loop to realize constant voltage charging. The effect is shown in Fig 11.

The working state of the CLLC resonant converter is more complicated, so the extended description function method is used to simplify the nonlinear state equation of its model, and linearization is performed. Finally, the transfer function of the CLLC resonant converter switching frequency to output voltage is obtained as follows [19]:

$$G_{vf}(s)=K_{vf}\frac{\frac{1}{s^{2}C_{r1}}-L_{r1}+\frac{n^{2}L_{r2}-\frac{n^{2}}{s^{2}C_{r2}}}{s^{3}L_{m}C_{r1}}}{{\left\{(\frac{1}{sC_{r1}}+sL_{r1})+(n^{2}sL_{r2}+\frac{n^{2}}{sC_{r2}}+\frac{8n^{2}}{\pi^{2}}R_{0})(1+L_{r1}+\frac{1}{s^{2}L_{m}C_{r1}})\right\}}^{2}}$$ (26)

Here, *R*_0_ is the load resistance, and the expression of *K*_vf_ is as follows:

$$K_{vf}=\frac{8n^{2}R_{0}U_{in}}{\pi^{2}}$$ (27)

**Fig 12:**
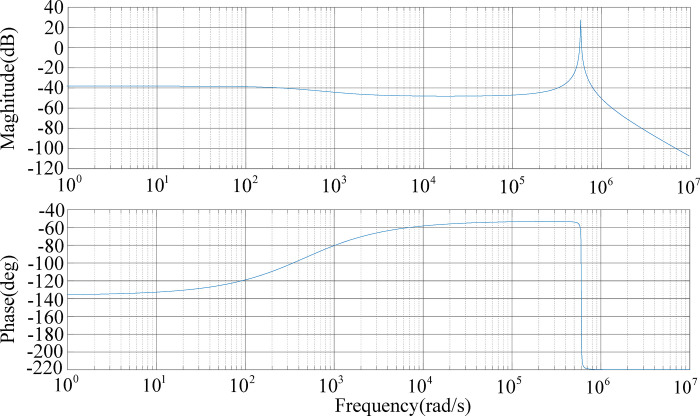
Bode plot of system transfer function without the PI controller.

The Bode plot of the system transfer function without the PI controller is shown in Fig 12. The gain of the system at the low-frequency end is low, and the cut-off frequency is too small.

Since the system is a Type 0 system before being corrected, it needs to be compensated to increase the system level so that the system drops at a slope of -20 dB/dec and crosses the 0 line, thereby increasing the phase angle margin of the system and improving the stability of the system.

The PI controller transfer function expression is as follows:

$$C(s)=K_{p}+\frac{K_{i}}{s}$$ (28)

After adding the PI controller, its closed-loop transfer function expression is as follows:

$$G(s)=\frac{C(s)K_{vf}(s)}{1+C(s)K_{vf}(s)}=\frac{K_{p}+\frac{K_{i}}{s}}{K_{p}+\frac{K_{i}}{s}+\frac{\pi^{2}{\left\{(\frac{1}{sC_{r1}}+sL_{r1})+(n^{2}sL_{r2}+\frac{n^{2}}{sC_{r2}}+\frac{8n^{2}}{\pi^{2}}R_{0})(1+L_{r1}+\frac{1}{s^{2}L_{m}C_{r1}})\right\}}^{2}}{8n^{2}R_{0}U_{in}(\frac{1}{s^{2}C_{r1}}-L_{r1}+\frac{n^{2}L_{r2}-\frac{n^{2}}{s^{2}C_{r2}}}{s^{3}L_{m}C_{r1}})}}$$ (29)

Carry on the double closed-loop PI parameter tuning to Eq (29), obtain the voltage outer loop, and the current inner loop controller expression as follows:

$${\mathrm{PI}}_{v}(s)=\frac{0.05\times (1+1.2\times 10^{3}s)}{s}$$ (30)

$${\mathrm{PI}}_{i}(s)=\frac{0.025\times (1+5\times 10^{3}s)}{s}$$ (31)

**Fig 13:**
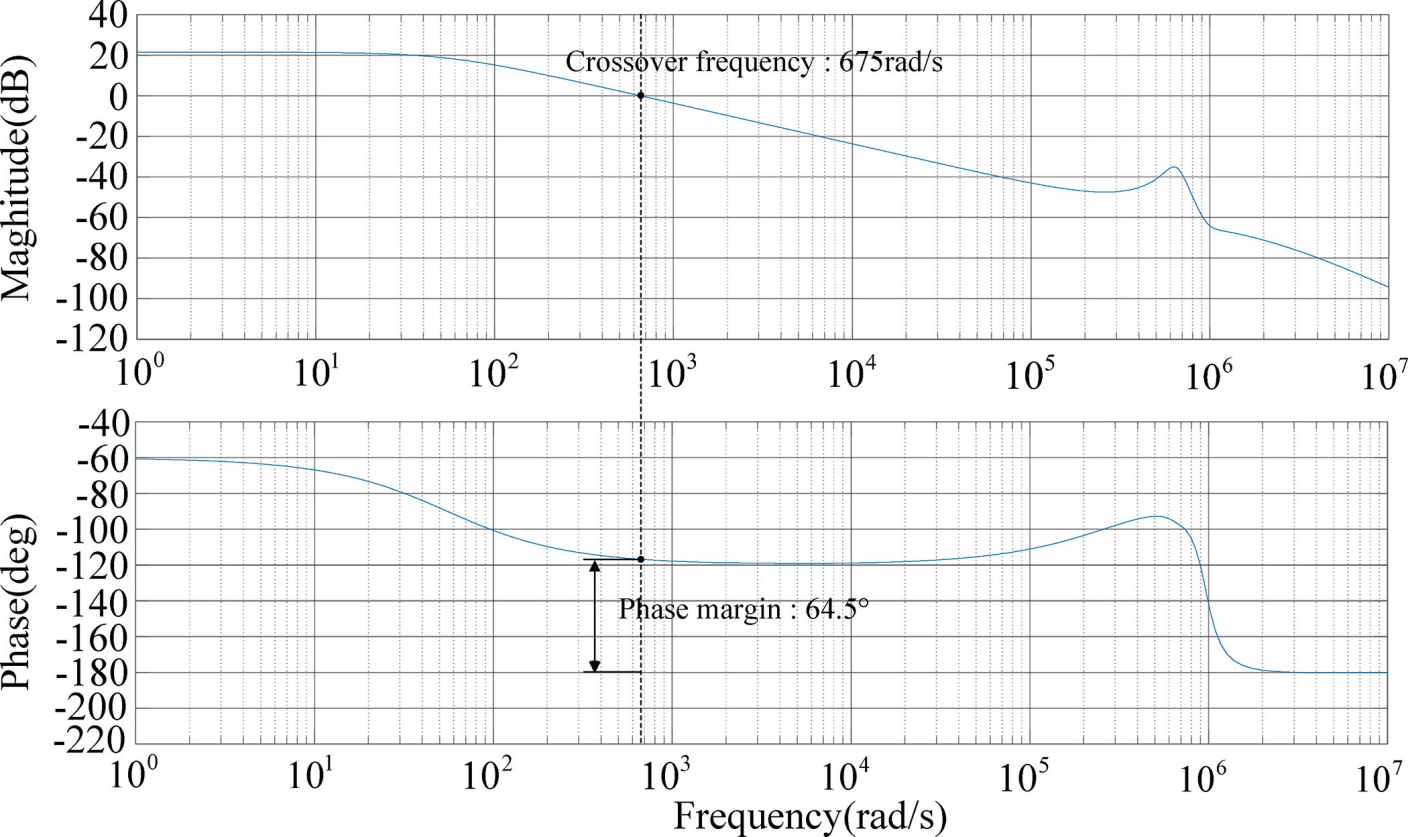
Bode plot of system transfer function added to the PI controller.

The Bode plot of the system transfer function after PI correction is shown in Fig 13. The crossover frequency is 675 rad/s and the phase margin is 64.5°. This system has a relatively stable mid-frequency band width and has strong anti-interference ability against high-frequency signals.

## 6 System simulation

**Fig 14:**
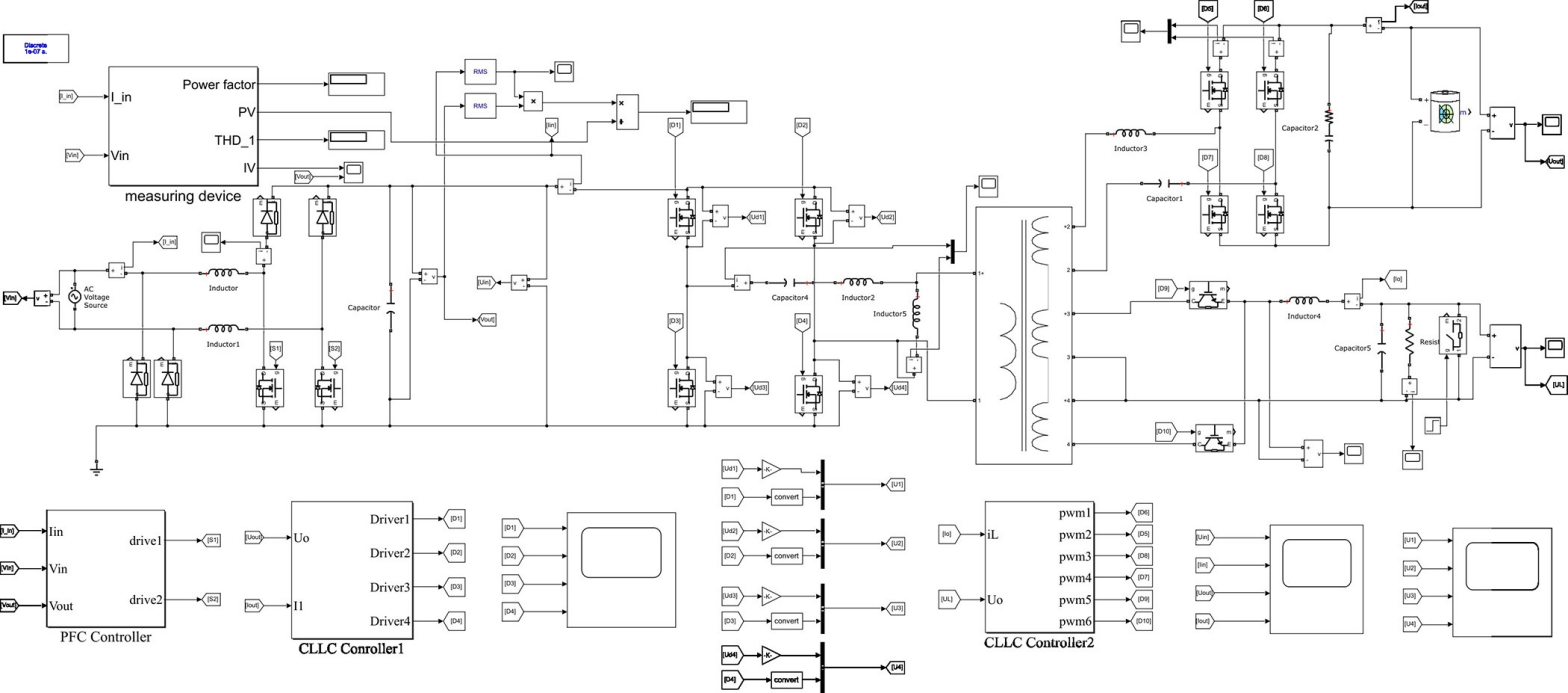
Simulation model of dual output port vehicle charging system.

According to the system parameters and control strategy, a simulation model of a dual-output port on-board charging system is built, as shown in Fig 14.

### 6.1 PFC simulation waveform

**Fig 15:**
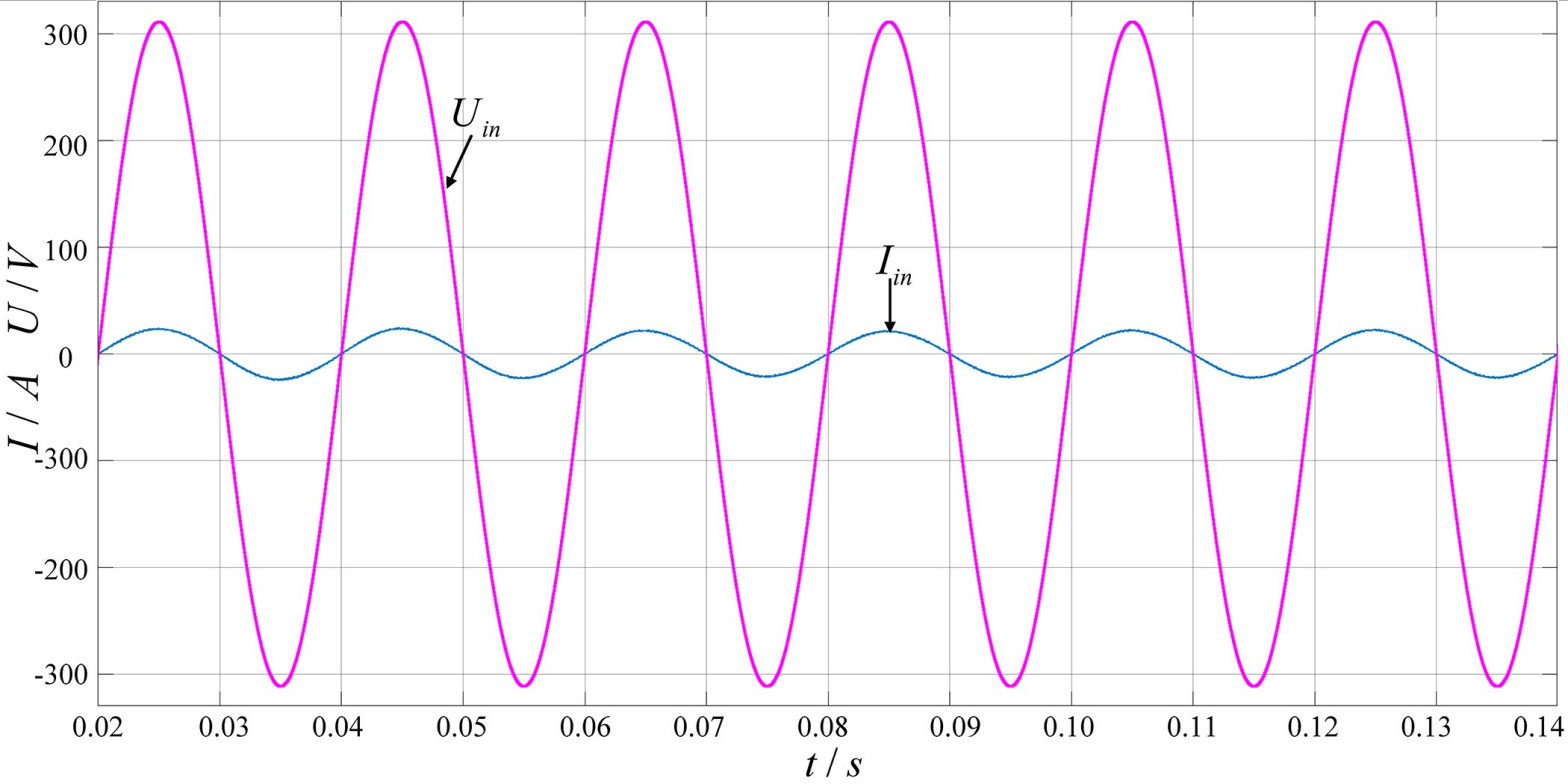
PFC input voltage and current waveform.

**Fig 16:**
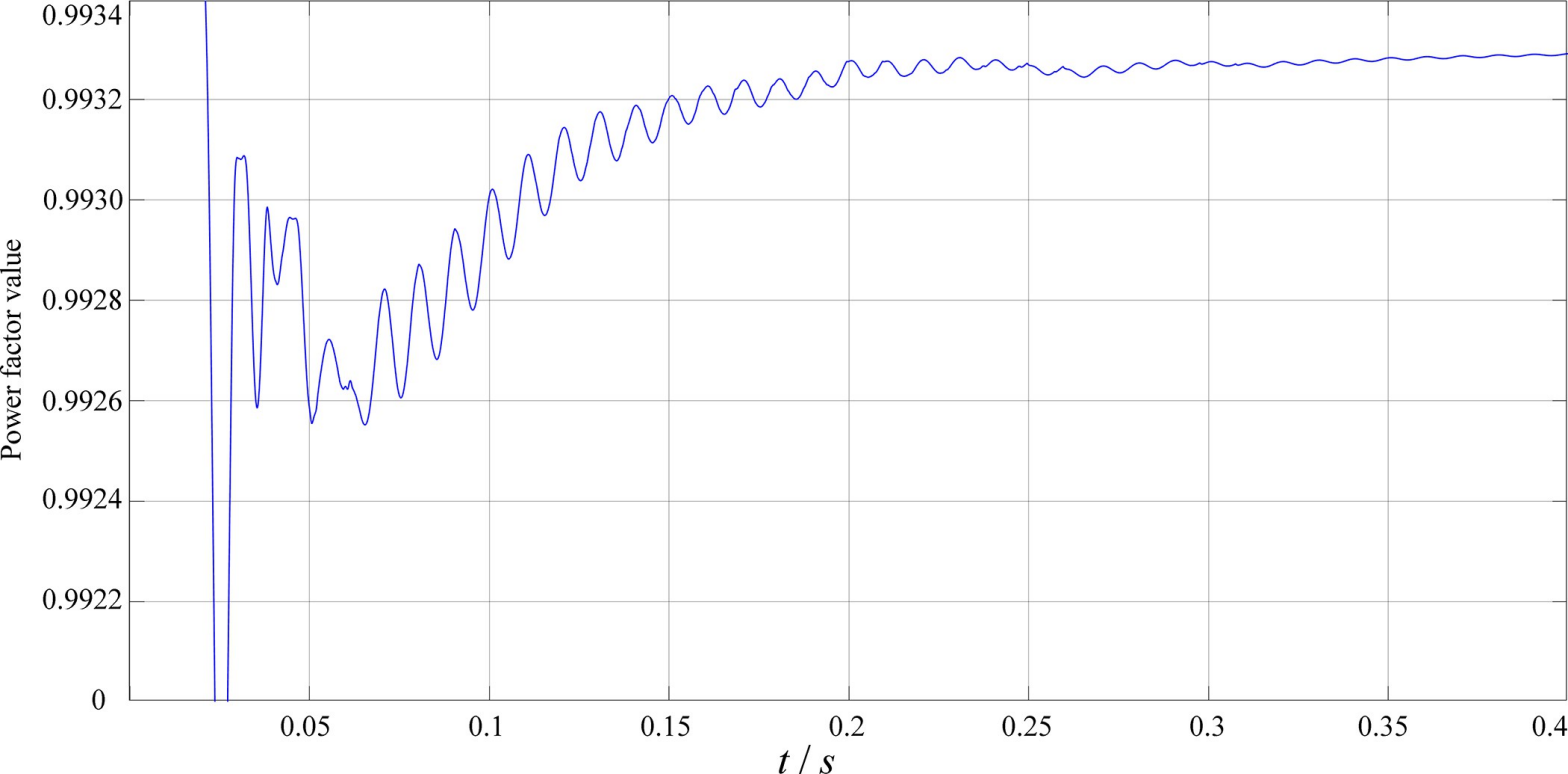
Power factor value.

Fig 15 shows the PFC input voltage and current waveforms. It can be seen from the figure that after adopting the dual closed-loop control strategy based on the average current method in the simulation, the input current of the circuit is a sine wave in the same phase with the input voltage (single-phase 220V AC). Fig 16 shows the power factor value, it can be seen that the power factor is greater than 0.99.

**Fig 17:**
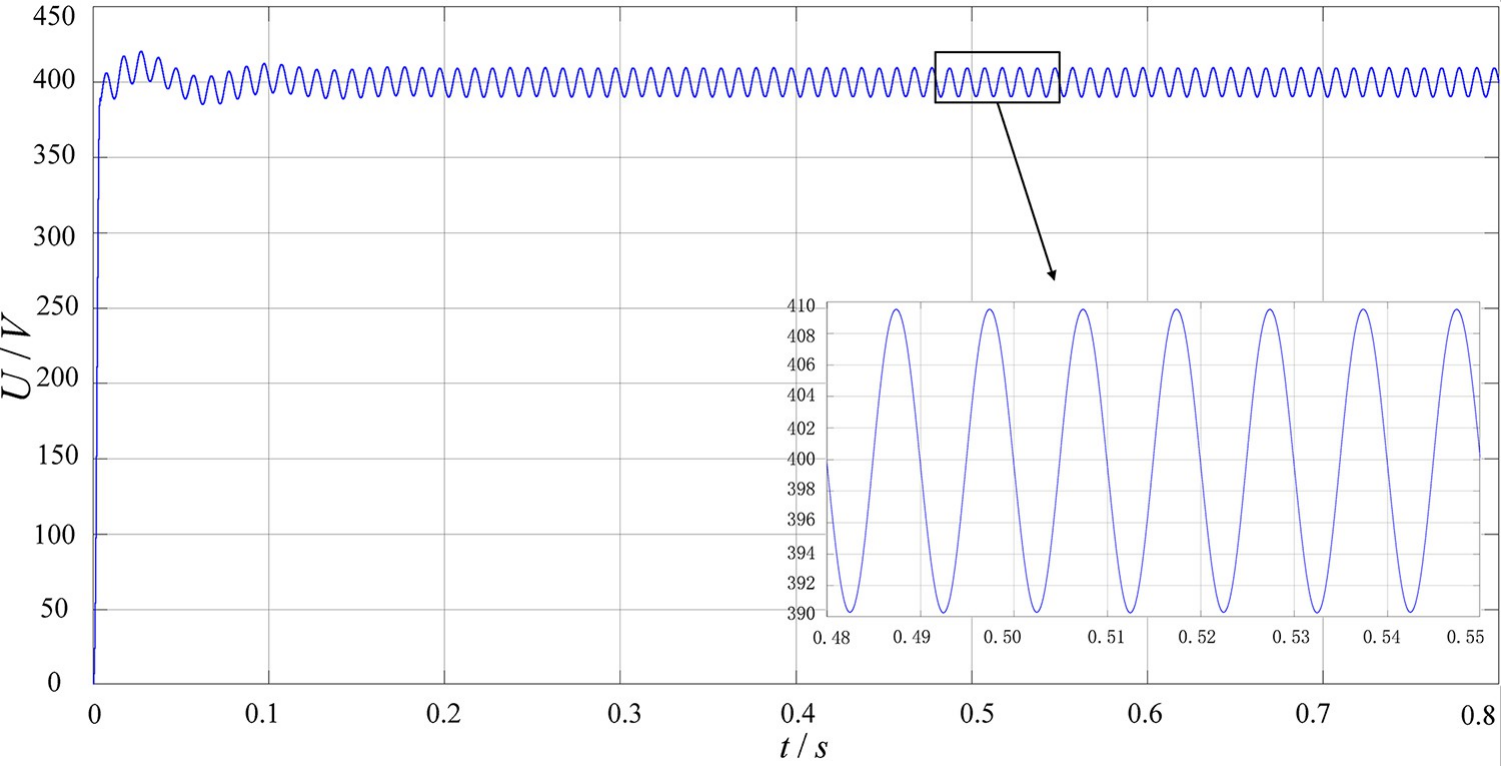
PFC output voltage waveform.

Fig 17 shows the PFC output voltage waveform. It can be seen from the figure that the output DC bus voltage can be stabilized at about 400V and its ripple coefficient is less than 2.5%.

**Fig 18:**
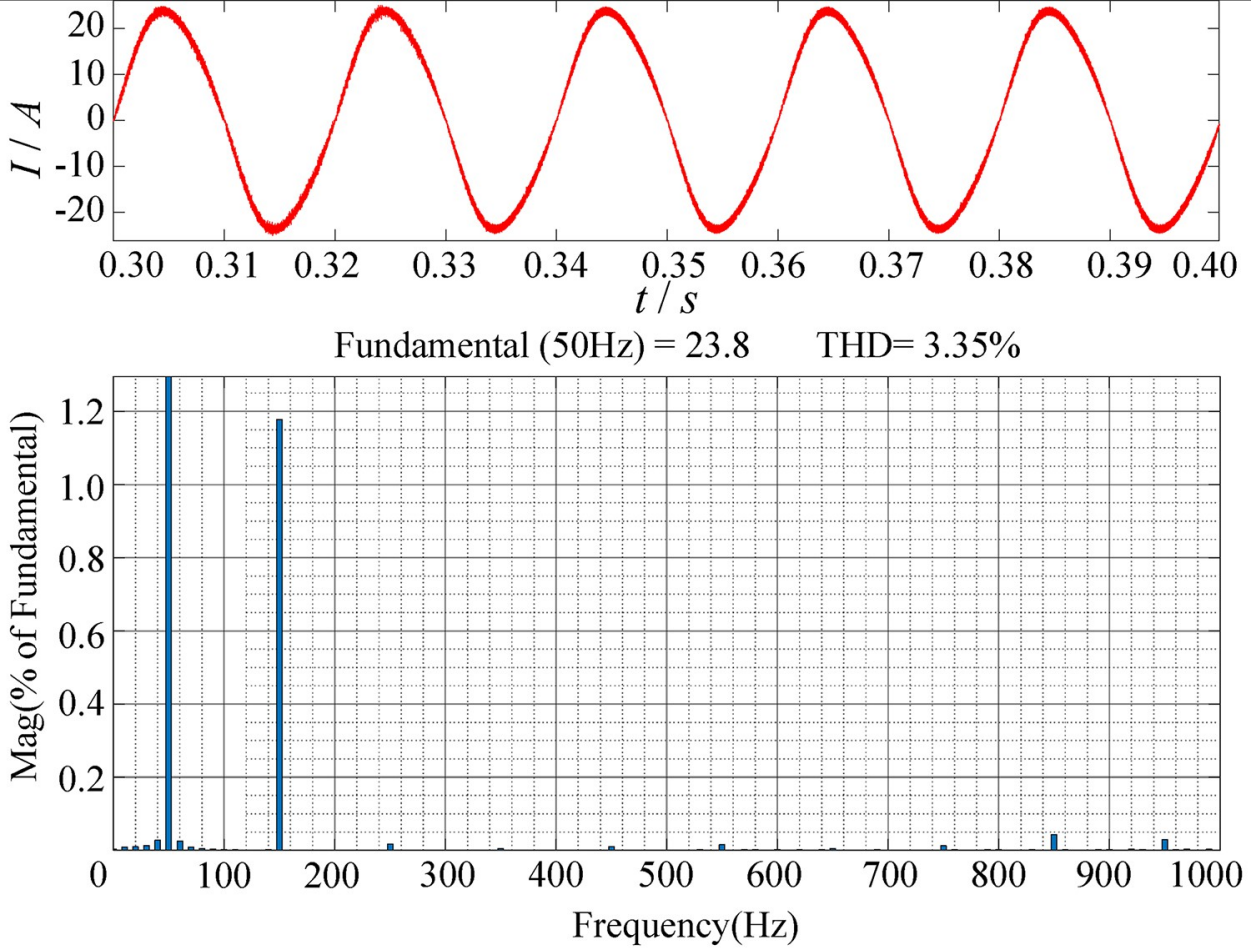
Diagram of the harmonic content of the PFC input current.

Fig 18 is a diagram of the harmonic content of the PFC input current. From the figure, it can be seen that the THD of the input current is 3.35%, the content of harmonics above the third is relatively low, and the suppression effect on harmonic interference is obvious.

### 6.2 Simulation waveform of the primary-side circuit

**Fig 19:**
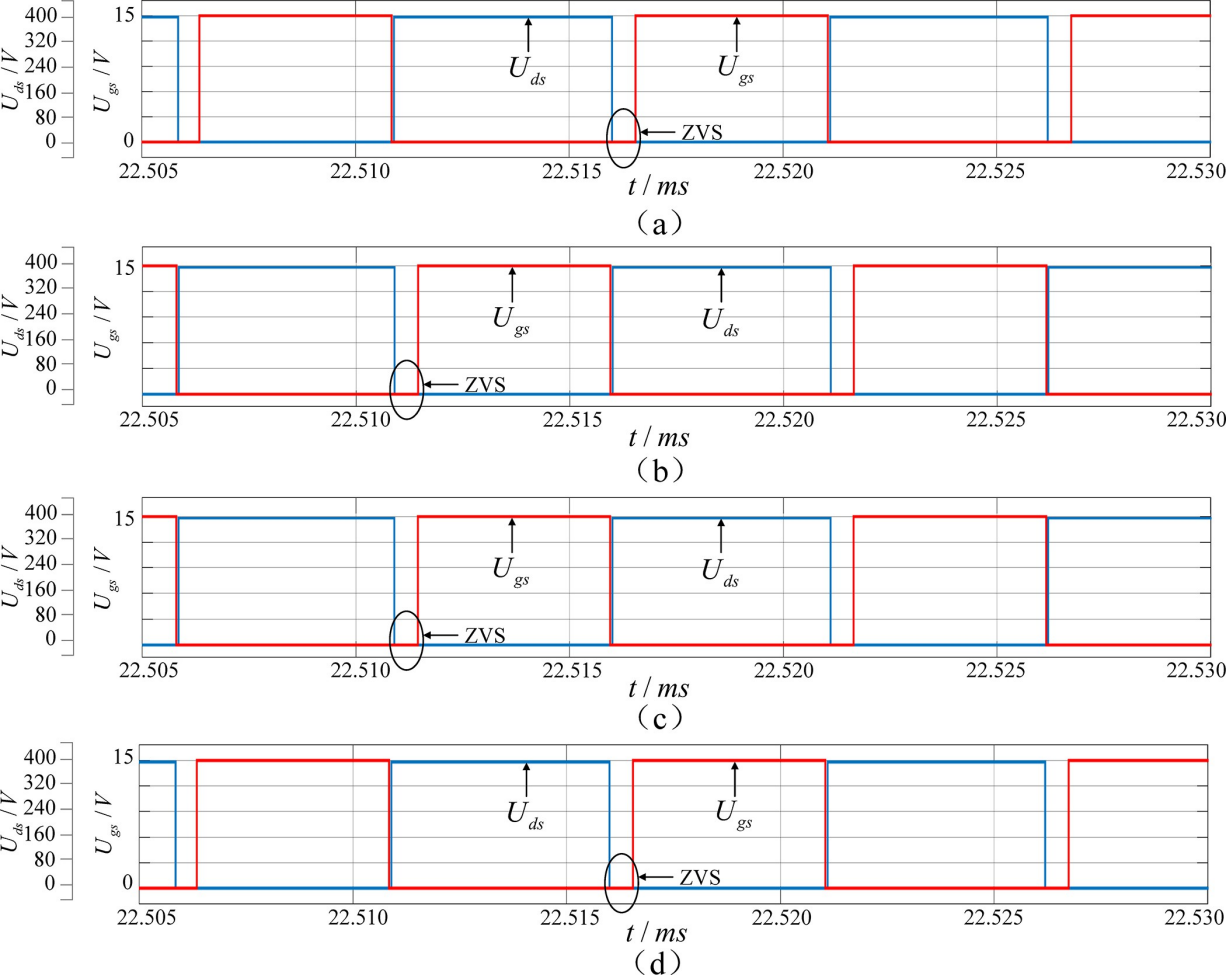
Operating waveforms of power switches *Q*_1_ (a), *Q*_2_ (b), *Q*_3_ (c) and *Q*_4_ (d) on the primary side.

Fig 19 shows the operating waveforms of the primary-side power switches *Q*_1_~*Q*_4_ when the system operates in parked charging mode. Before the drive signal arrives, the drain-source voltage of the power switches has been pulled to zero potential by the body diode to realize ZVS and reduce the switching loss.

**Fig 20:**
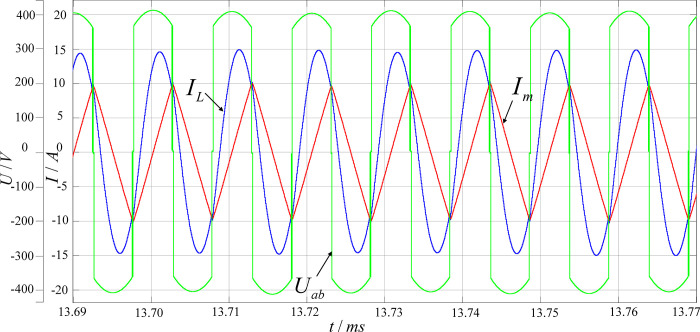
Voltage and current waveforms of the resonant cavity at full load.

Fig 20 shows the waveforms of resonant current *I*_L_, excitation current *I*_m_, and the center point voltage *U*_ab_ of the primary side at full load. When the output voltage is 400V, the switching frequency is basically equal to the resonant frequency (100kHz). At this time, the magnetizing inductor does not participate in resonance and presents a triangular wave shape, while the primary-side resonant capacitor and inductor both participate in resonance. Furthermore, the resonant current *I*_L_ is approximately a sine wave.

### 6.3 Simulation waveform of high-voltage charging circuit

**Fig 21:**
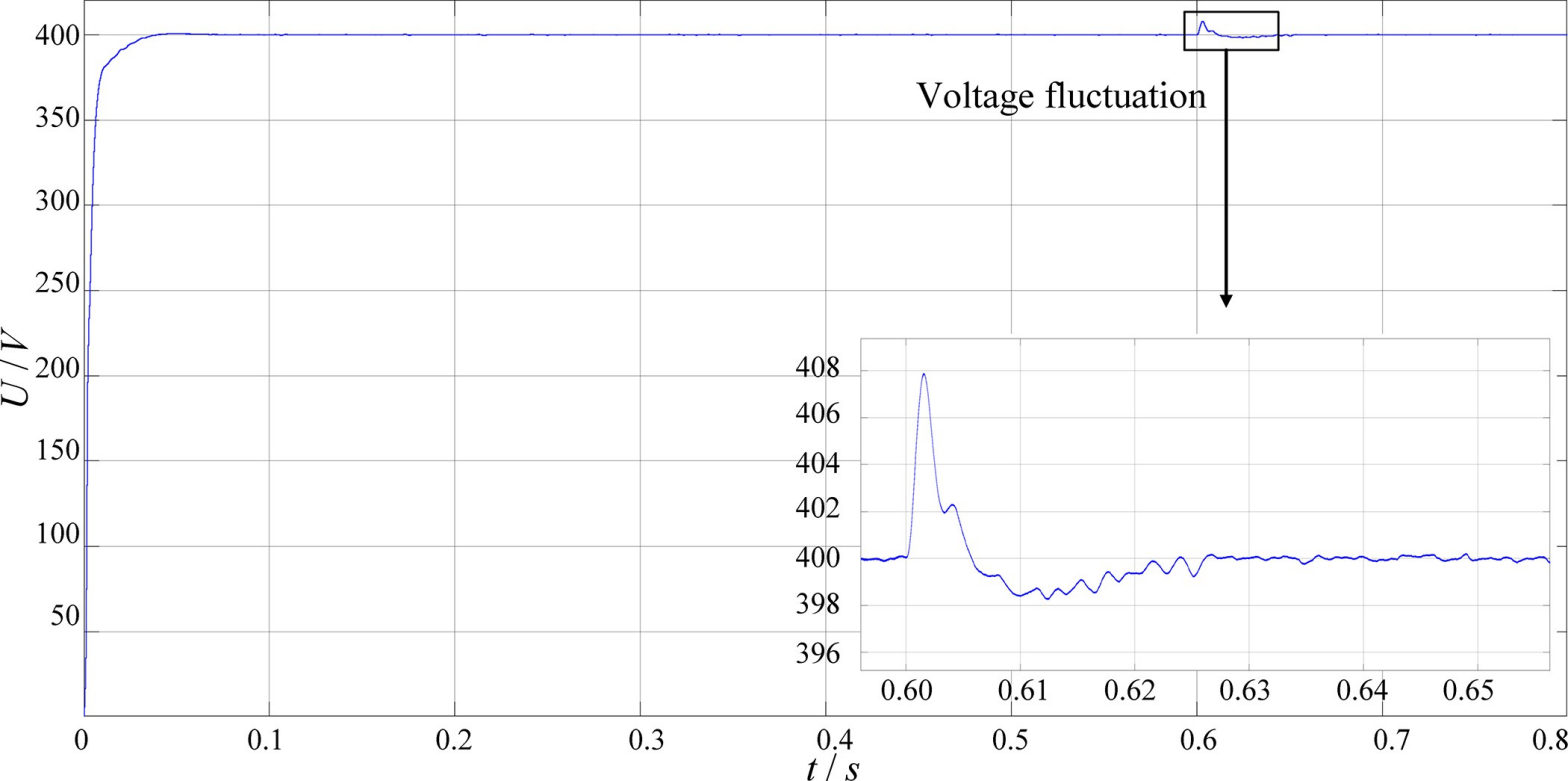
The output voltage waveform of the high-voltage charging circuit (400V).

Fig 21 shows the output voltage waveform when the output voltage is 400V and the equivalent load is 50 Ω. It can be seen from the figure that the output voltage is stable at about 400V at the time of steady state. Set a 5% input voltage fluctuation at 0.6s and the output voltage returns to stability after a short period of oscillation. It can be seen that the fluctuation range is less than 2%, and it has a strong anti-interference ability.

**Fig 22:**
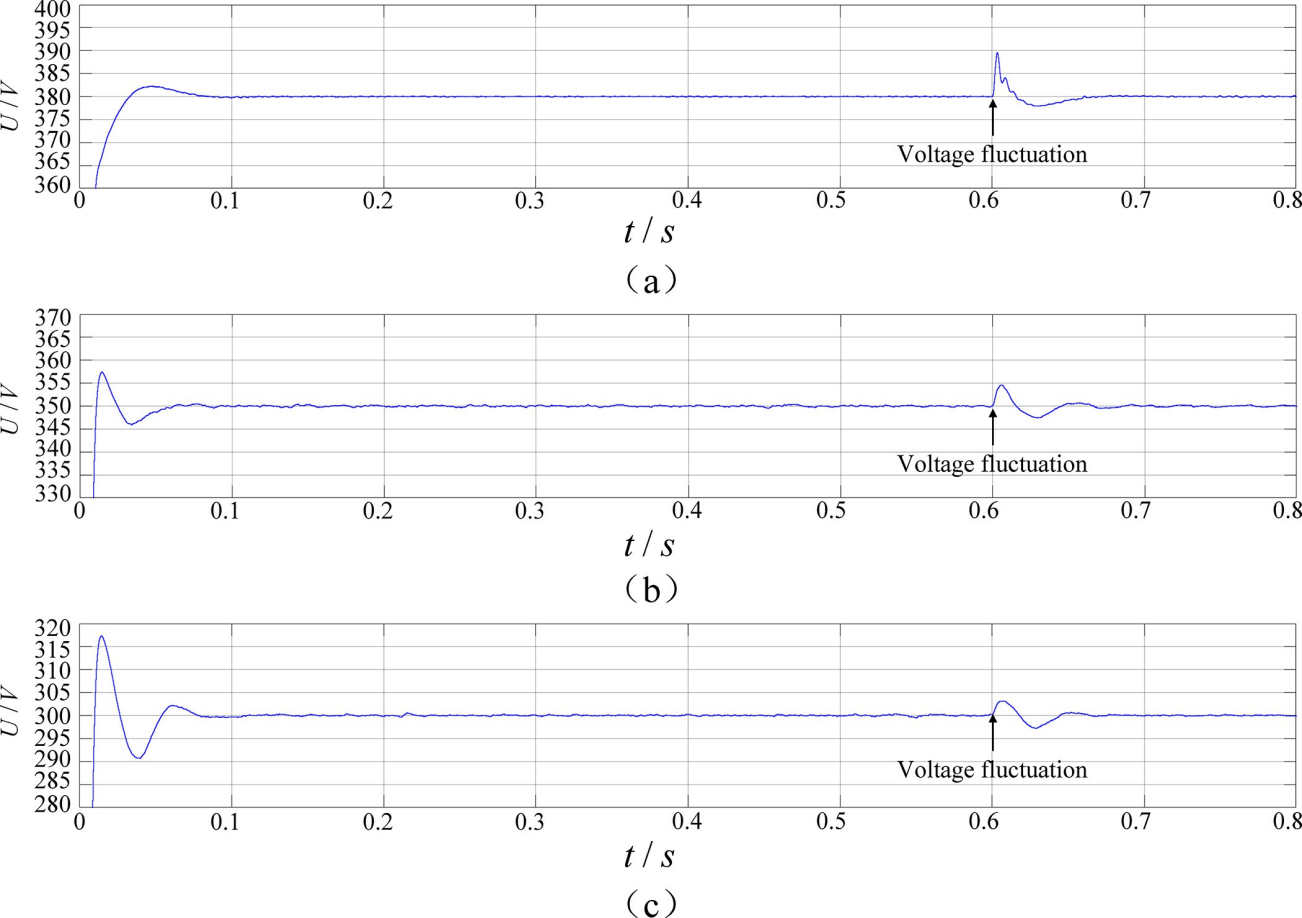
The output voltage waveform when the output voltage is 380V(a), 350V(b) and 300V(c).

Fig 22 shows the equivalent load 50 in parked charging mode, and the output voltage waveforms at 380V, 350V, and 300V. It can be seen that the output voltage is stable near the present voltage value in the steady state, reaching the design output voltage range, the input voltage fluctuation and oscillation are small and it has a wide and stable voltage gain characteristic.

**Fig 23:**
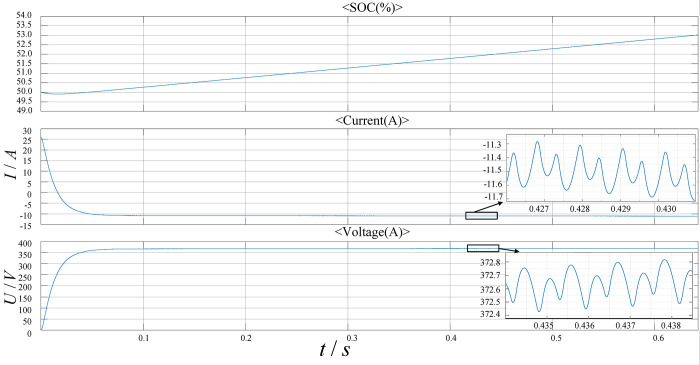
The charging process of the high voltage power battery.

Fig 23 shows the charging process of the high-voltage power battery. It can be seen from the figure that during the charging process, the state of charge (SOC) of the battery rises steadily and the stable charging current is about 11A. When the bus voltage is low, the battery first undergoes a short discharge and then starts charging. The charging voltage finally stabilizes at 370V.

### 6.4 Simulation waveform of the low-voltage charging circuit

**Fig 24:**
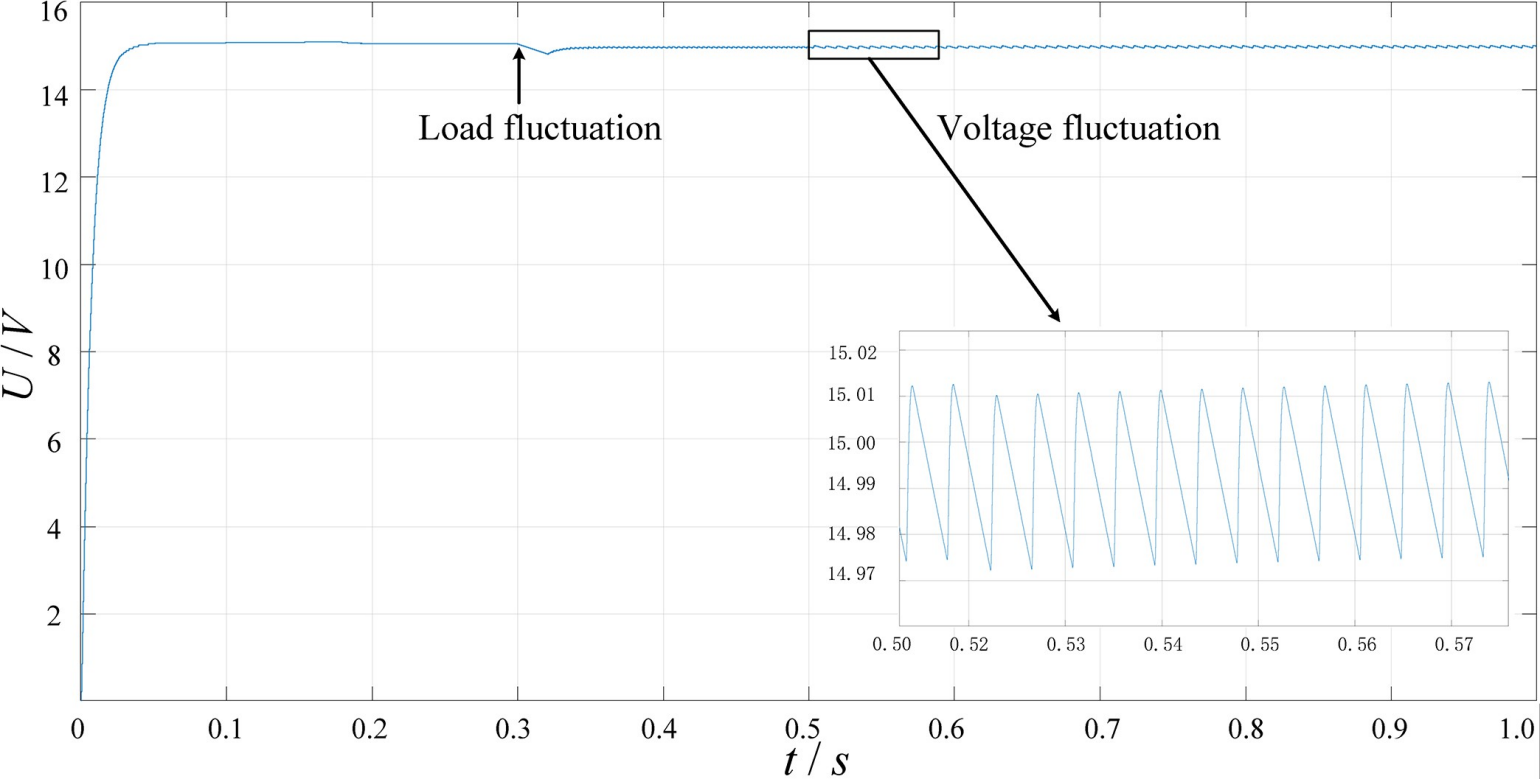
The waveform of the output voltage of the low-voltage charging circuit.

Fig 24 shows the output voltage waveform of the low-voltage charging circuit under full load conditions and the voltage is stable at 15V at the time of steady state. Set a 50% load fluctuation in 0.3s and its output voltage quickly returns to a stable value after a short drop. Set a 10% input voltage fluctuation in 0.6s and the output voltage will return to stability after a slight oscillation. Furthermore, the voltage ripple will be less than 0.1%.

## 7 Experimental verification

**Fig 25:**
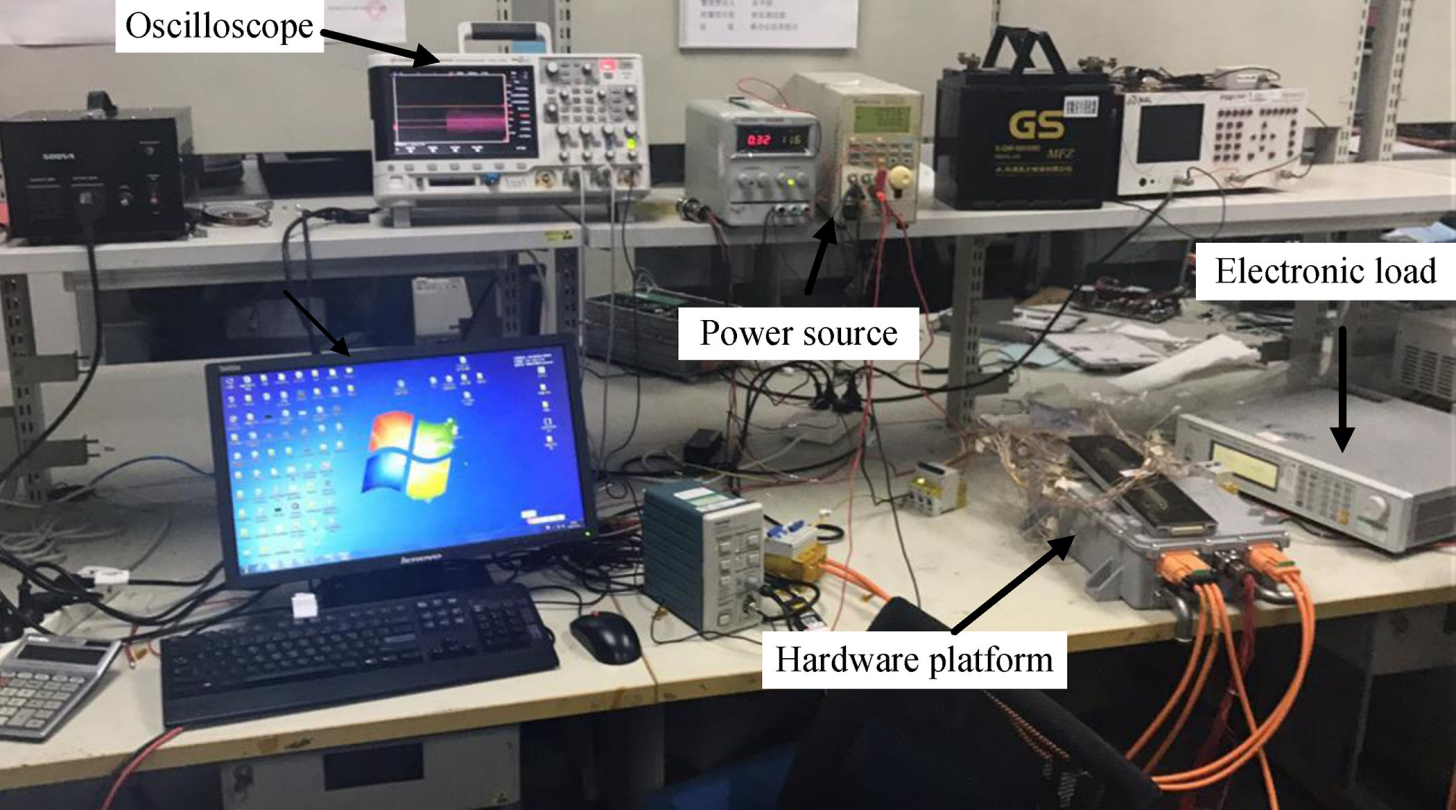
The experimental prototype of a dual-output port on-board charging system.

Based on the simulation model, an experimental prototype of a dual-output port on-board charging system was built. The experimental setup is illustrated in (Fig 25).

**Fig 26:**
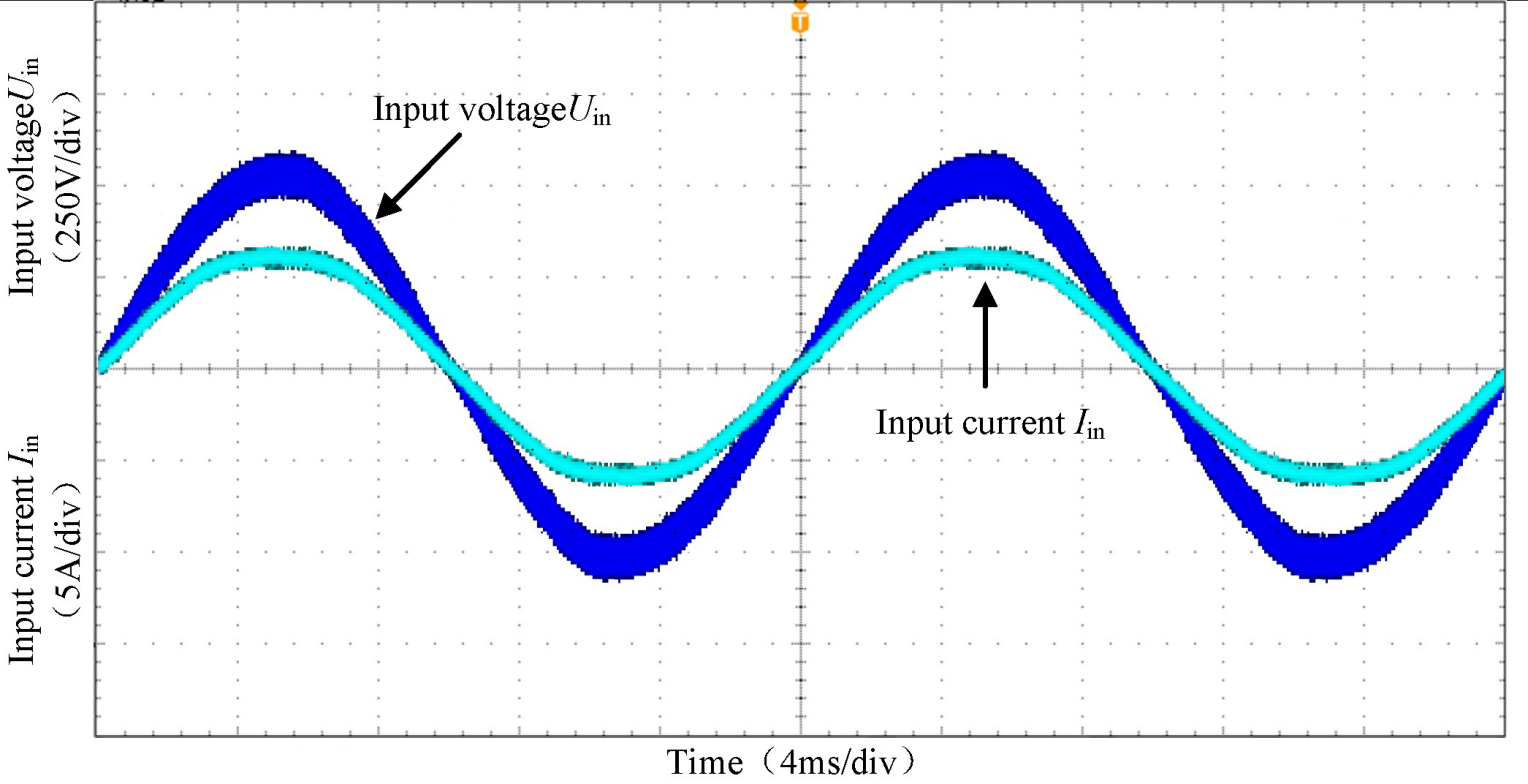
The input voltage and current waveforms of PFC.

Fig 26 shows the input voltage and current waveforms of PFC under full load condition. It can be seen from the figure that the designed dual-diode bridgeless PFC circuit can realize the power factor correction function of the input current.

**Fig 27:**
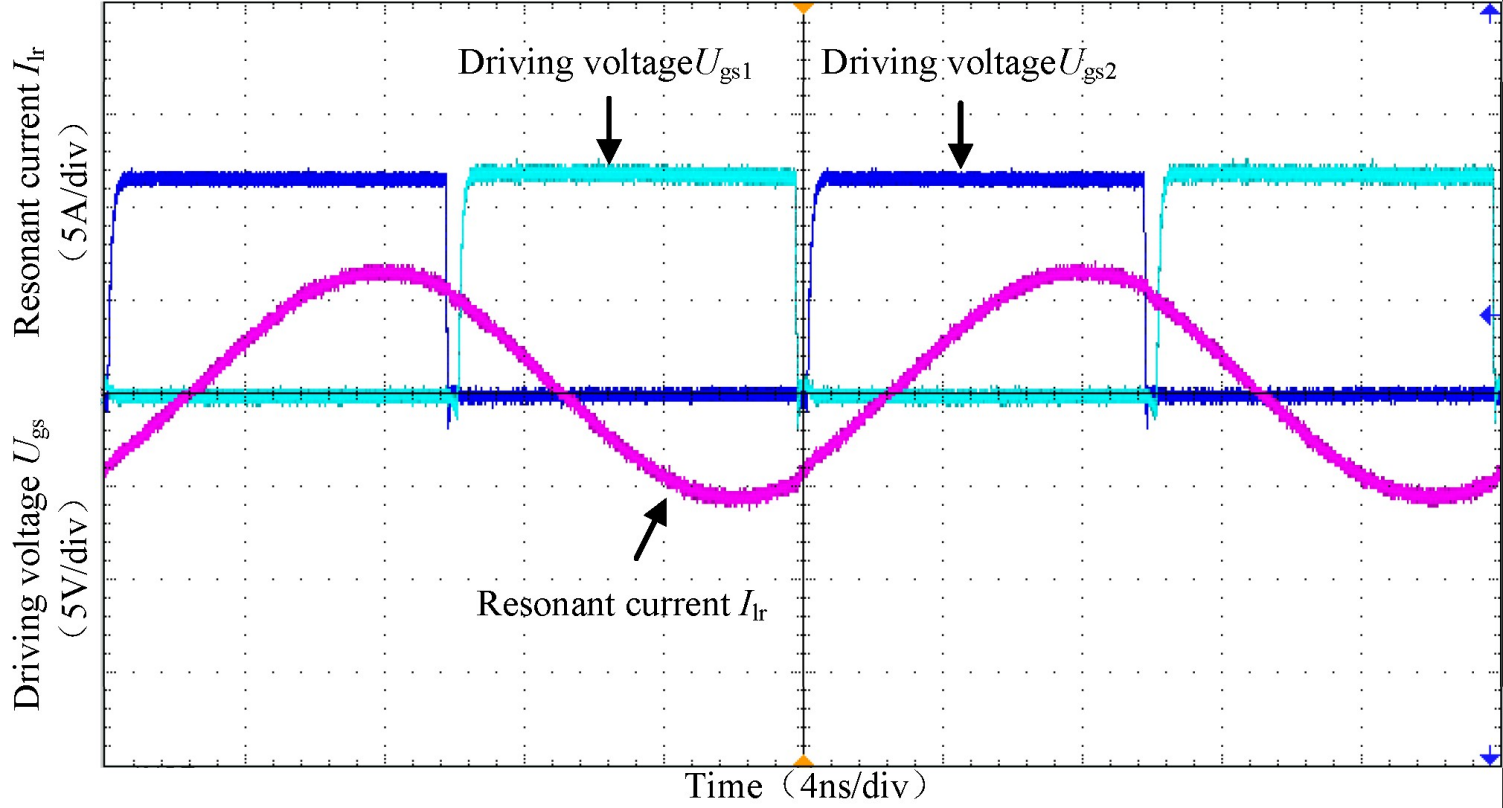
The waveforms of driving voltages and resonant current.

Fig 27 shows the waveforms of driving voltages *U*_gs_1 and *U*_gs_2 and resonant current I_*Lr*_ of two complementary MOS tubes with the full bridge of the original side at full load. When the switching frequency is equal to the resonant frequency, both the original edge-side resonant capacitor and inductor participate in resonance, and the resonant current *I*_Lr_ is approximately a sine wave.

**Fig 28:**
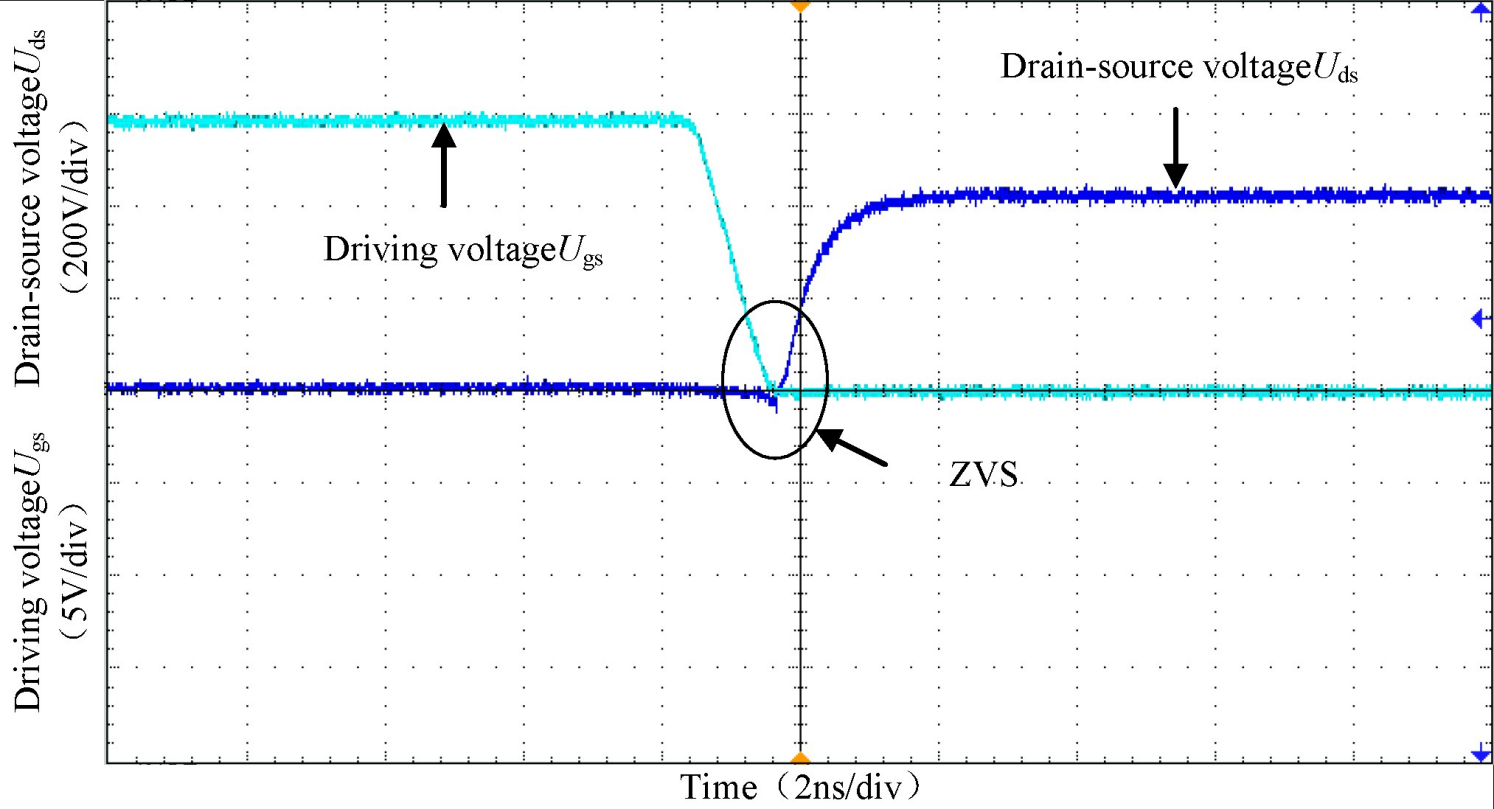
The waveforms of the full-bridge switch.

Fig 28 shows the waveform of the full-bridge switch on the original side. When the driving voltage *U*_gs_ arrives, the drain-source voltage *U*_ds_ of the switch has been pulled to zero potential by its body diode, realizing the ZVS of the power switch of the original side circuit.

**Fig 29:**
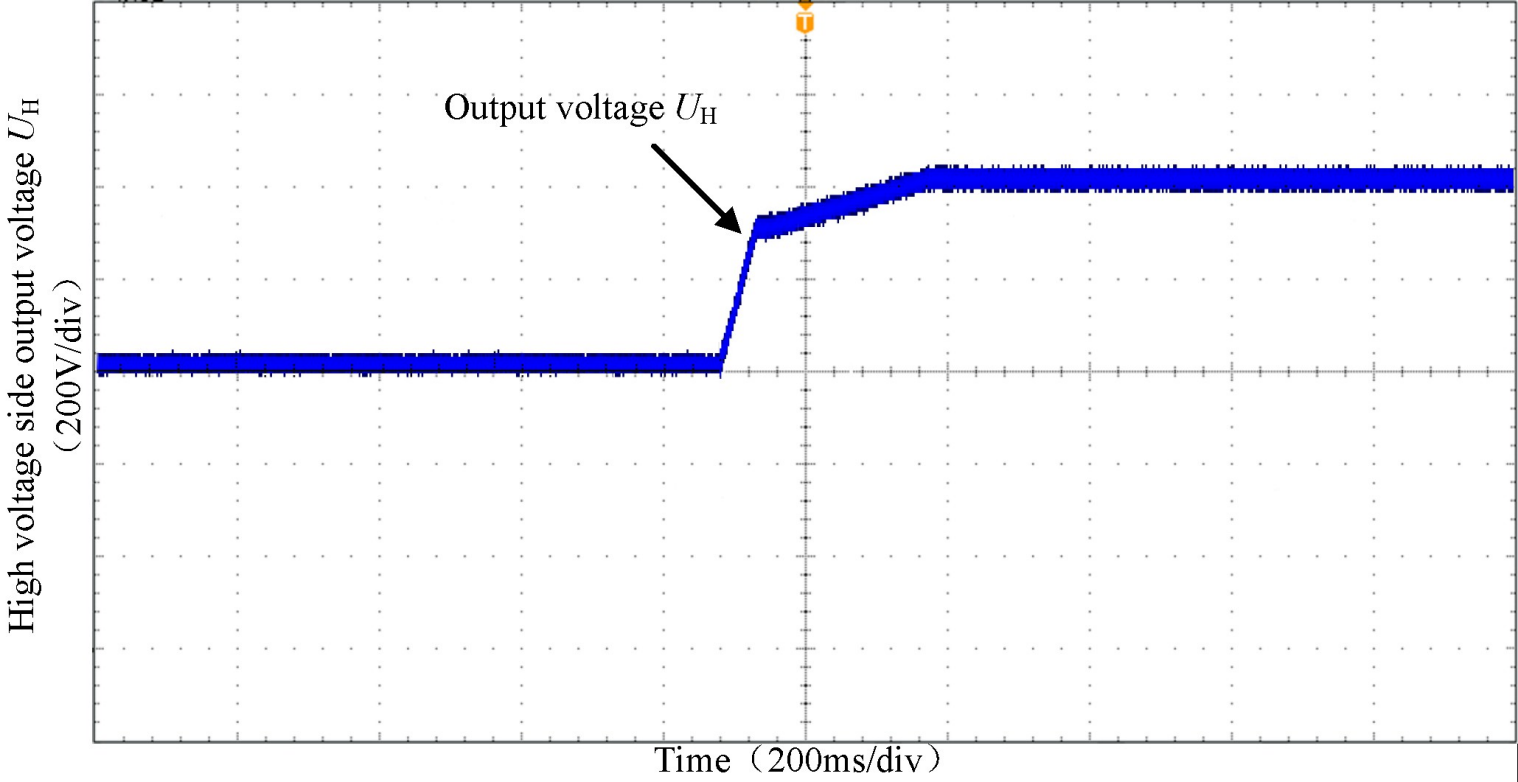
The output voltage waveform of the high-voltage side.

**Fig 30:**
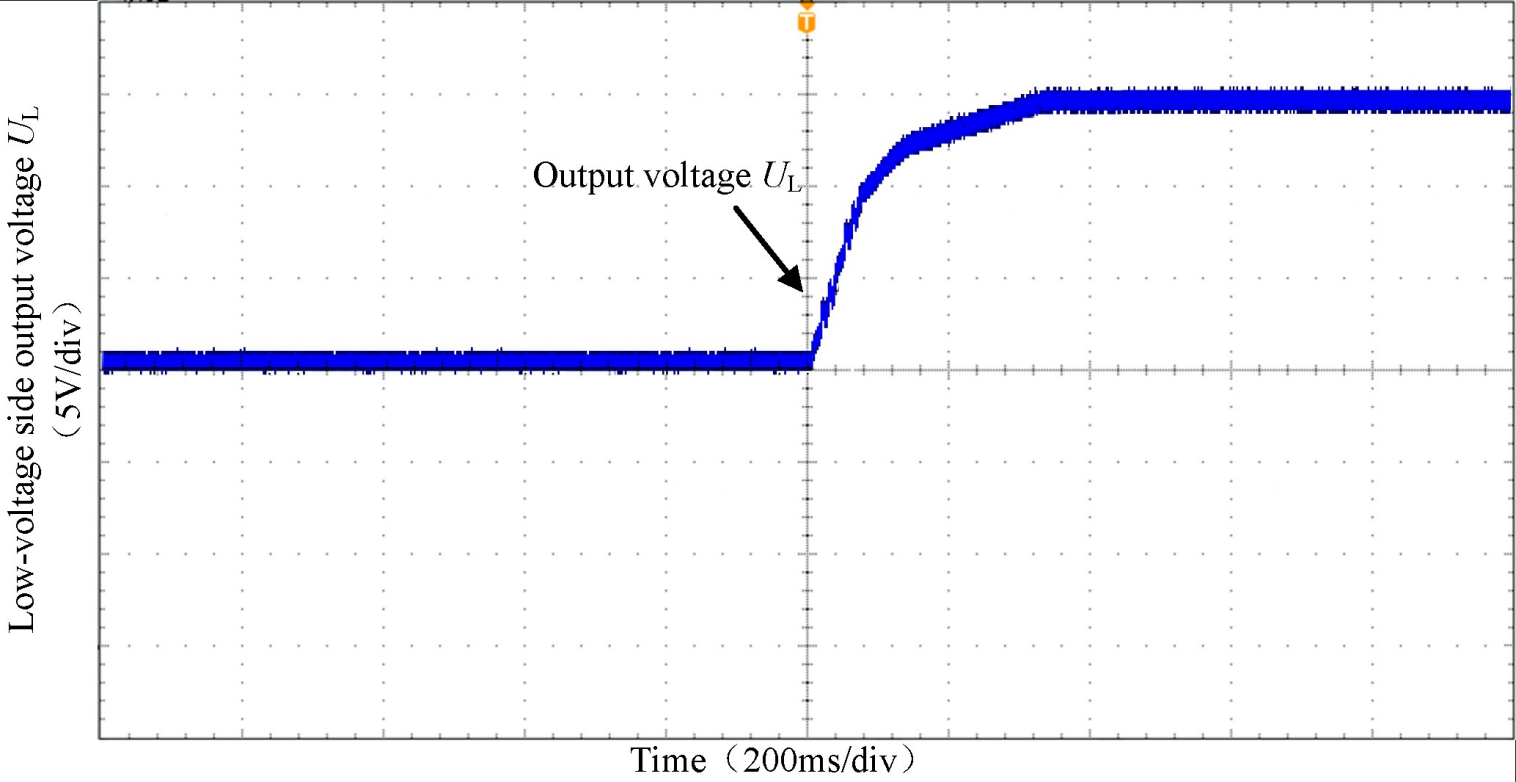
The output voltage waveform of the low-voltage side.

Figs 29 and 30 show the output voltage waveform of the high-voltage side and low-voltage side of the converter under full load condition. It can be seen that under the double closed-loop PI control strategy, the voltage rise is relatively stable and reaches the peak value within 0.5s.

**Fig 31:**
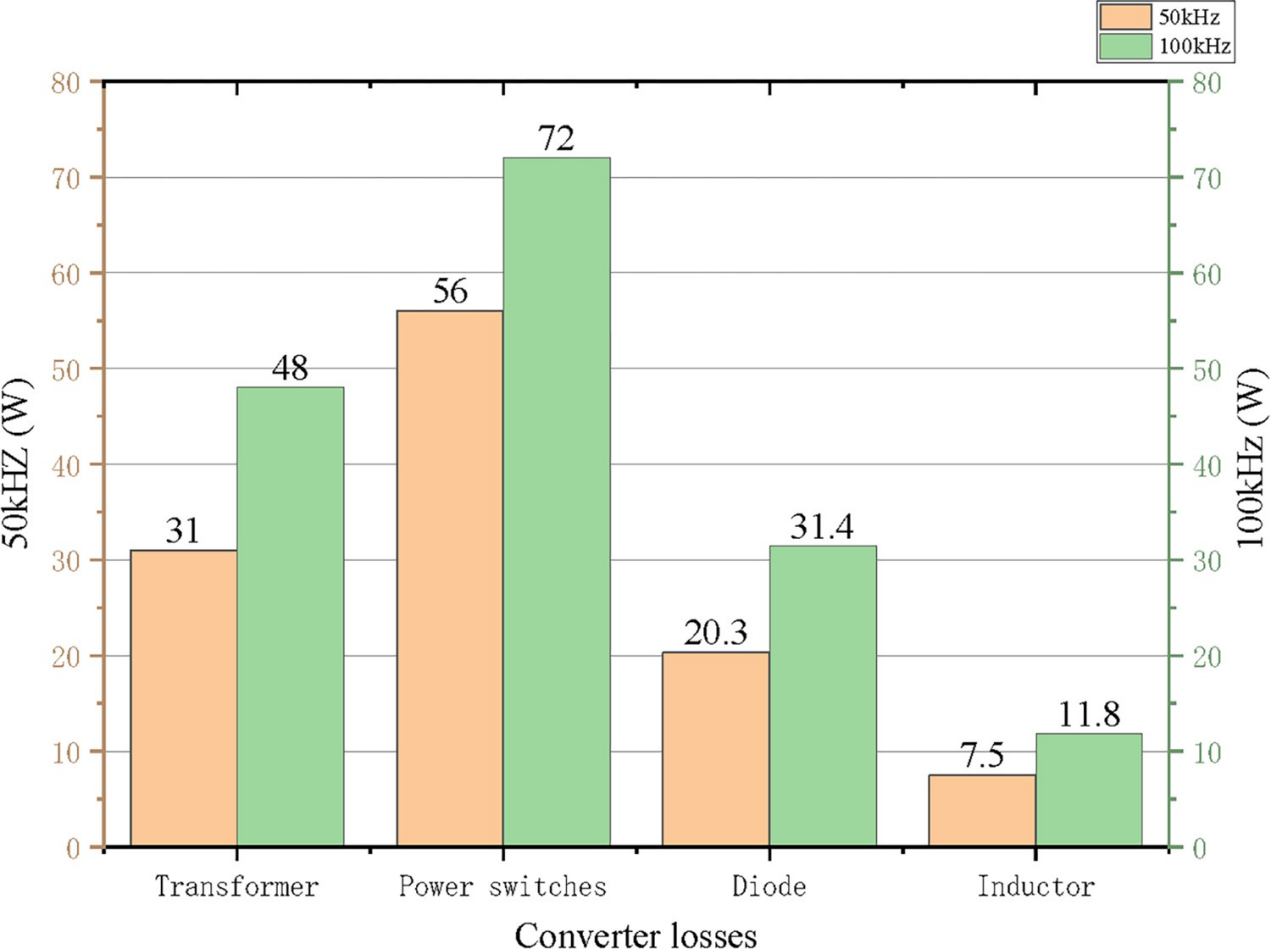
The diagram of component loss.

Fig 31 shows the component loss comparison diagram of the converter operating at two operating frequencies under full load condition. It can be seen that the total loss of the converter operating at 0.5 times resonant frequency (50kHz) is 114.8W, and the working efficiency is 96.52%. At the resonant frequency point (100kHZ), the total loss of the converter is 163.2W, and the working efficiency is 95.05%, which meets the design requirements.

**Fig 32:**
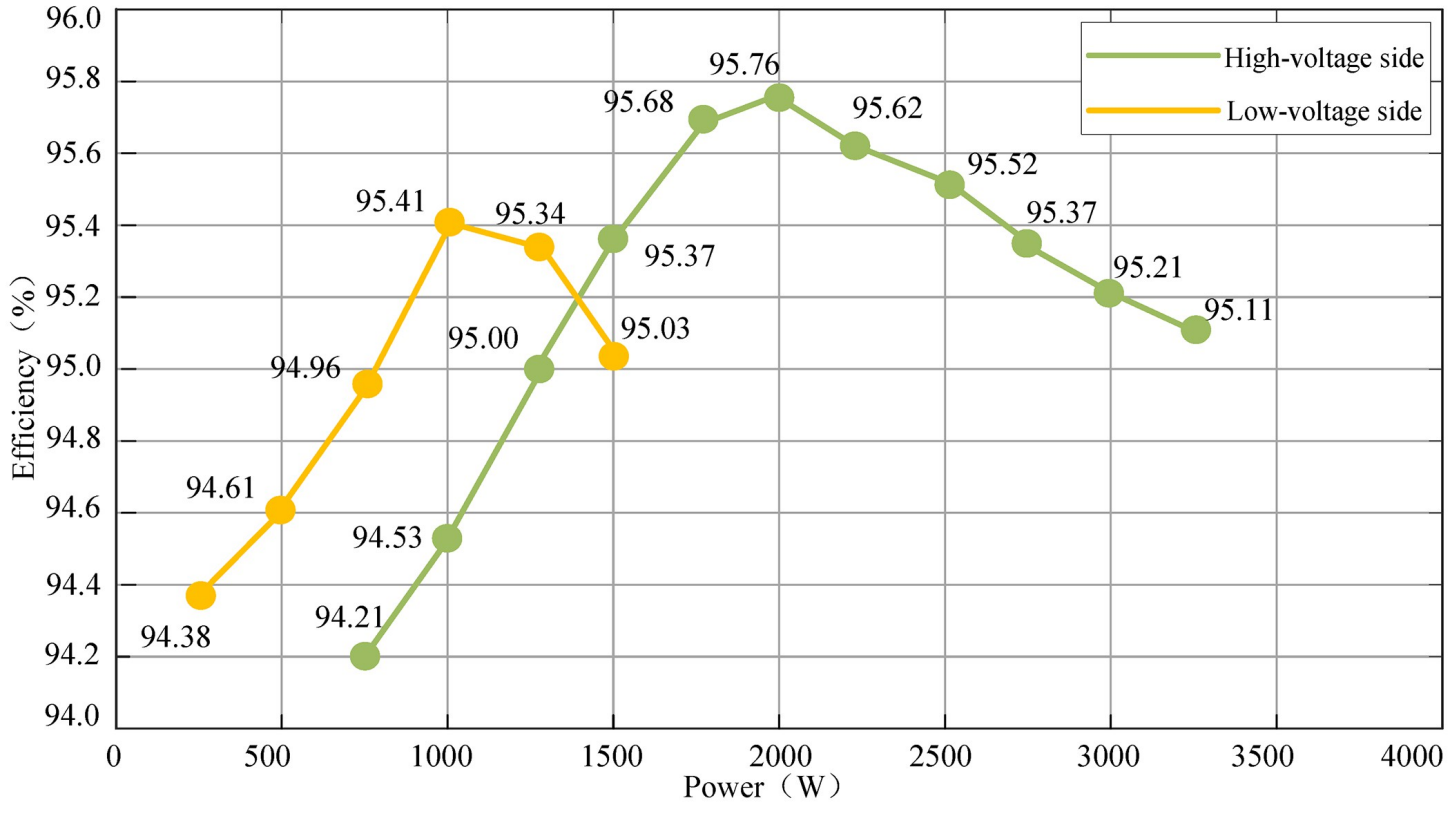
The diagram of charge operation efficiency.

Fig 32 shows the comparison diagram of the charging operation efficiency of the converter. It can be seen that the charging efficiency of the high-voltage side circuit under half-load condition is 95.37%, the full-load efficiency is 95.11%, and the maximum working efficiency is 97.86%. Under the condition of half-load, the charging efficiency of the low-voltage side circuit is 94.96%, the full load efficiency is 95.03%, and the maximum working efficiency is 95.76%, which meets the design requirements.

## 8 Conclusion

This paper designs a dual-output port on-board charging system based on the CLLC resonant converter. The front stage of the system uses dual-diode bridgeless PFC and the latter stage uses dual-output port CLLC resonant conversion that integrates high and low voltage charging circuits on a transformer. Based on the fundamental wave analysis method, an equivalent model of the CLLC resonant converter is established, the gain characteristics are analyzed, the key parameters of the system are calculated, and the system control strategy is analyzed. Experimental results show that the on-board charging system can achieve power factor correction, has a high power factor, can charge high-voltage power battery packs and low-voltage batteries in the full voltage range, and can achieve soft switching in both modes.

## References

[1] Wang B, Dehghanian P, Wang S, Mitolo M. Electrical Safety Considerations in Large-Scale Electric Vehicle Charging Stations. IEEE Transactions on Industry Applications. 2019; 55(06): 6603–6612.

[2] Zhou K, Yuan C, Sun D, Jin N, Wu X. Parameter adaptive terminal sliding mode control for Full-Bridge DC-DC converter. PLOS ONE. 2021; 16(2): e0247228. doi: 10.1371/journal.pone.0247228 33630901 PMC7906401

[3] Etezadi-Amoli M, Choma K, Stefani J. Rapid-Charge Electric-Vehicle Stations. IEEE Transactions on Power Delivery. 2010; 25(03): 1883–1887.

[4] Yao L, Li D, Liu L. An improved large signal model of full-bridge LLC converter. PLOS ONE. 2018; 13(10): e0205904. doi: 10.1371/journal.pone.0205904 30339681 PMC6195276

[5] Abid M, Ahmad F, Ullah F, Habib U, Nawaz S. High voltage DC power supply with power factor correction based on LLC resonant converter. PLOS ONE. 2020; 15(9): e0239008. doi: 10.1371/journal.pone.0239008 32956410 PMC7505463

[6] Goswami R, Wang S. Investigation and Modeling of Combined Feedforward and Feedback Control Schemes to Improve the Performance of Differential Mode Active EMI Filters in AC–DC Power Converters. IEEE Transactions on Industrial Electronics. 2019; 66(08): 6538–6548.

[7] Wu H, Zhang Y, Jia Y. Three-Port Bridgeless PFC-Based Quasi Single-Stage Single-Phase AC–DC Converters for Wide Voltage Range Applications. IEEE Transaction on Industry Applications. 2018; 65(07): 5518–5528.

[8] Zeng J, Zhang G, Yu S S, Zhang B, Zhang Y. LLC resonant converter topologies and industrial applications-A review. Chinese Journal of Electrical Engineering. 2020; 6(03): 73–84.

[9] Mallik A, Khaligh A. A High Step-Down Dual Output Nonisolated DC/DC Converter with Decoupled Control. IEEE Transaction on Industry Applications. 2018; 54(01): 722–731.

[10] Chen G, Jin Z, Deng Y, He X, Qing X. Principle and Topology Synthesis of Integrated Single-Input Dual-Output and Dual-Input Single-Output DC–DC Converters. IEEE Transaction on Industry Applications. 2018; 65 (05): 3815–3825.

[11] Prabhakaran P, Agarwal V. Novel Four-Port DC–DC Converter for Interfacing Solar PV–Fuel Cell Hybrid Sources with Low-Voltage Bipolar DC Microgrids. IEEE Journal of Emerging and Selected Topics in Power Electronics. 2020; 8(02): 1330–1340.

[12] Wu H, Sun K, Chen R, Hu H, Xing Y. Full-Bridge Three-Port Converters with Wide Input Voltage Range for Renewable Power Systems. IEEE Transaction on Power Electronics, 2012; 34(12): 11940–11951.

[13] Dao N D, Lee D, Phan Q D. High-Efficiency SiC-Based Isolated Three-Port DC/DC Converters for Hybrid Charging Stations. IEEE Transaction on Power Electronics. 2020; 35(10): 10455–10465.

[14] Oluwasogo E S, Cha H. Self-Current Sharing in Dual-Transformer-Based Triple-Port Active Bridge DC–DC Converter with Reduced Device Count. IEEE Transaction on Power Electronics. 2021; 36(05): 5290–5301.

[15] Zhou k, Gu F C, Yang J C. LLC Circuit with Dual Output Port and Control Technique. Electric Machines and Control. 2022; 25(01): 17–26.

[16] Feng F, Wu F, Gooi H B. Impedance Shaping of Isolated Two-Stage AC-DC-DC Converter for Stability Improvement. IEEE Access. 2019; 7(06): 18601–18610.

[17] Zhao L, Pei Y Q, Liu X H, Fan W J, Du Y. Design Methodology of CLLC Resonant Converters for Electric Vehicle Battery Chargers. Proceedings of the Chinese Society for Electrical Engineering. 2020; 40(15): 4965–4977.

[18] Li G, Xia J, Wang K, Deng Y, He X, Wang Y. Hybrid Modulation of Parallel-Series LLC Resonant Converter and Phase Shift Full-Bridge Converter for a Dual-Output DC–DC Converter. IEEE Journal of Emerging and Selected Topics in Power Electronics. 2019; 7(02): 833–842.

[19] Zou S, Mallik A, Lu J, Khaligh A. Sliding Mode Control Scheme for a CLLC Resonant Converter. IEEE Transaction on Power Electronics. 2019; 34(12): 12274–12284.

